# Radical diffusion, not lifetime, determines the range of peroxidase-based proximity labelling

**DOI:** 10.1242/jcs.264887

**Published:** 2026-06-15

**Authors:** Samarpita Sen, Daniel St Johnston

**Affiliations:** The Gurdon Institute and Department of Genetics, University of Cambridge, Tennis Court Rd, Cambridge CB2 1QN, UK

**Keywords:** Proximity labelling, Epithelia, Membrane proteins, Apical–basal polarity, Diffusion, Basement membrane

## Abstract

Proximity labelling offers a powerful strategy for mapping the molecular composition in the vicinity of a protein of interest. Here, we employed APEX2- and HRP-mediated biotinylation in the *Drosophila* follicular epithelium to analyse the apical, lateral and basal membrane proteomes, using Cadherin99c (Cad99c), Fasciclin 3 (Fas3) and Nidogen (Ndg) as baits. We unexpectedly found that standard peroxidase-based labelling conditions produced a strong basal biotinylation signal, even when the tagged cargo localized apically or laterally. This arises from the long-range diffusion of phenoxy radicals, far exceeding the presumed ∼20 nm labelling radius. The basement membrane acts as a high-capacity sink for these radicals, owing to its abundance of electron-rich amino acids. Titrating the concentration of biotin-phenol or biotin-SS-tyramide or shortening reaction times restored spatially faithful labelling at both the plasma membrane and in intracellular compartments. Our results reveal that the distance over which proteins are labelled by peroxidase-based proximity labelling is not limited by the lifespan of the biotin radicals and can extend over many micrometres when radical production exceeds the number of reactive targets. This approach therefore requires careful optimization to avoid misleading spatial signatures.

## Teaser

**Summary:** Peroxidase proximity labelling in *Drosophila* epithelia shows unexpected long–range radical diffusion, and optimizing radical levels restores accurate spatial proteome mapping.

## INTRODUCTION

Cellular polarity enables cells to establish spatial asymmetry and to selectively localize proteins to defined subcellular domains, ensuring that specialized biochemical processes occur at the appropriate locations. This principle of spatial organization underlies epithelial apical–basal polarity. Epithelial cells form continuous, tightly packed layers that line the interface between the internal milieu of the organism and the external environment. This position confers the critical function of regulating molecular exchange across the tissue. To fulfil this role, epithelia display structurally and functionally distinct membrane domains: the apical surface faces the lumen, the lateral surface mediates cell–cell contacts, and the basal surface interfaces with the extracellular matrix (ECM) and the underlying connective tissue. These domains arise from the apical–basal polarization of epithelial cells and are maintained by the selective trafficking and retention of integral and peripheral membrane proteins to their appropriate membrane domains.

Many secreted and transmembrane proteins in epithelial cells are delivered to their correct destinations by polarized trafficking pathways that sort cargo-containing vesicles at the trans-Golgi network and subsequently target them to specific membrane domains. In mammalian systems, this process is often described using a binary apical versus basolateral framework. In contrast, studies in *Drosophila* have revealed that epithelial membrane organization is considerably more complex and occurs at a much finer spatial scale. In photoreceptor cells, for example, Crumbs (Crb), Rhodopsin (encoded by *ninaE*) and Eyes shut (Eys) are delivered to three distinct apical subdomains: Crumbs to the stalk membrane, Rhodopsin to the rhabdomere and Eys to the inter-rhabdomeral space (Gurudev et al., 2014; Laffafian and Tepass, 2019; Satoh et al., 2005; Wang et al., 2014). Similarly, in the *Drosophila* follicular epithelium, ECM components are secreted to spatially distinct basal regions, with Nidogen (Ndg) and Collagen IV deposited at the leading and trailing edges of migrating follicle cells, respectively (Isabella and Horne-Badovinac, 2016; Richens et al., 2025). These observations suggest that the traditional apical–basolateral dichotomy is insufficient to capture the true molecular complexity of epithelial membranes in *Drosophila*.

Despite these advances, the protein composition of individual membrane domains in most *Drosophila* epithelial tissues remains poorly defined and only a limited number of cargoes have been characterized in detail. We therefore sought to systematically characterize the proteomic landscape of each membrane domain in the *Drosophila* follicular epithelium through proximity labelling to identify new cargoes that are enriched within them.

**Fig. 1.**
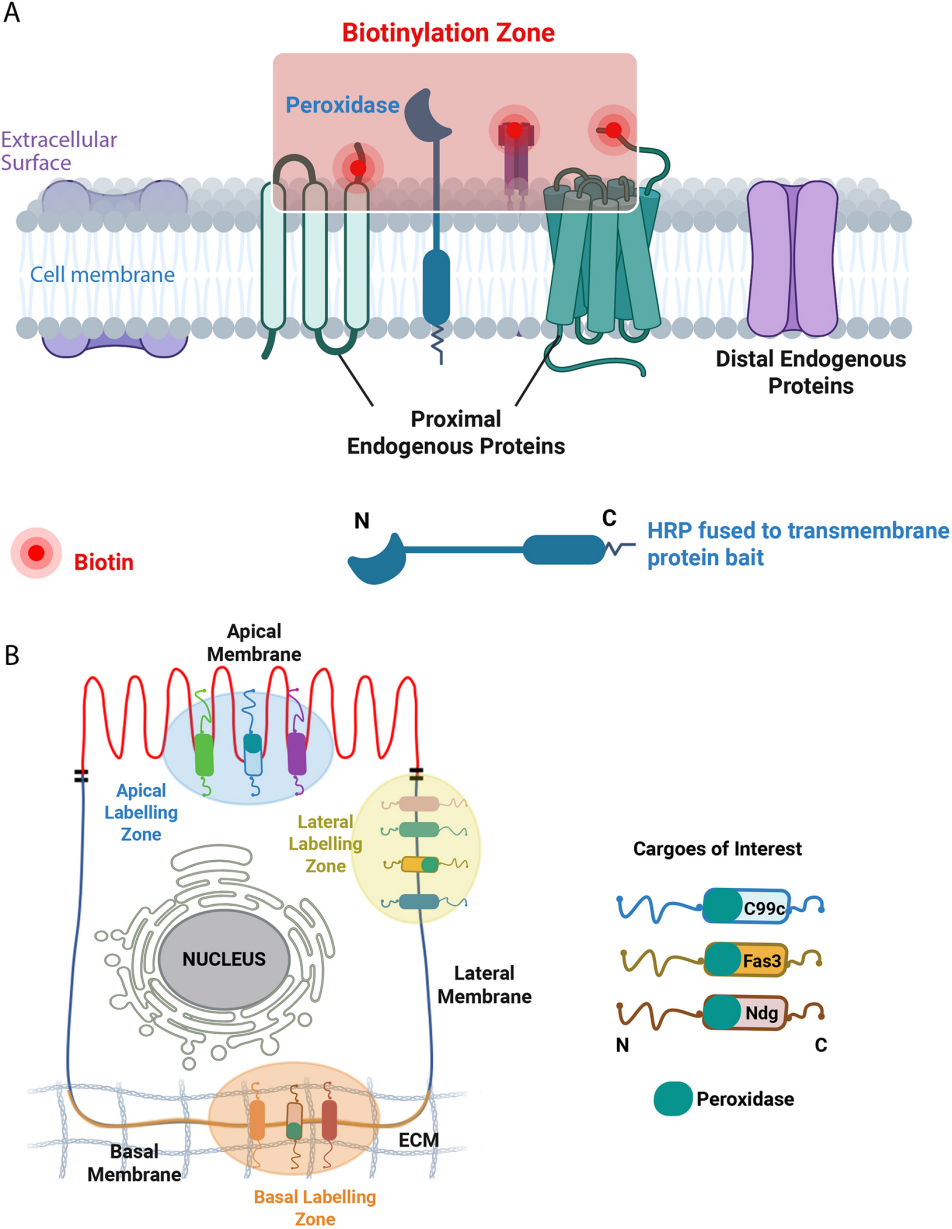
**Strategy for spatially resolved proximity labelling of membrane proteomes in *Drosophila* follicle cells.** (A) A diagram illustrating peroxidase-mediated proximity labelling at the cell surface. A transmembrane bait protein tagged with a peroxidase at its extracellular N-terminus catalyses the generation of short-lived biotin-phenoxy radicals, resulting in covalent biotinylation of proteins located within the immediate vicinity of the bait (biotinylation zone), whereas more distal membrane proteins remain unlabelled. Created in BioRender by Sen, S., 2026. https://BioRender.com/utxvbri. This figure was sublicensed under CC-BY 4.0 terms. (B) Overview of the experimental design for comparative mapping of apical, lateral and basal membrane proteomes in follicle cells. Peroxidase-tagged apical (Cadherin99c; Cad99c or C99c), lateral (Fasciclin 3 or Fas3) and basal (Nidogen or Ndg) cargoes define discrete labelling zones corresponding to the three polarized membrane domains. Biotinylated proteins within each domain are subsequently enriched and identified by mass spectrometry. Created in BioRender by Sen, S., 2026. https://BioRender.com/lvsippy. This figure was sublicensed under CC-BY 4.0 terms.

Peroxidase-mediated proximity labelling has recently emerged as a powerful approach for identifying proteins residing within ∼10–20 nm of a peroxidase-tagged bait (Guo et al., 2023; Rhee et al., 2013). In this technique, engineered peroxidases such as ascorbate peroxidase 2 (APEX2) or horseradish peroxidase (HRP) catalyse the oxidation of biotin-phenol (BP) derivatives to generate short-lived phenoxy radicals that covalently biotinylate nearby proteins, thereby enabling the capture of local proteomes with high spatial and temporal resolution (Fig. 1A) (Liu et al., 2018; Roux et al., 2012).

Here, we employed APEX2- and HRP-mediated proximity biotinylation to profile the apical, lateral and basal membrane proteomes of *Drosophila* follicle cells using three well-characterized cargoes as baits (Fig. 1B). Cadherin99c (Cad99c), our apical bait, is a member of the cadherin superfamily that promotes the formation of microvilli-like structures in follicle cells and is secreted apically (Schlichting et al., 2006). Fasciclin 3 (Fas3), our lateral cargo, is an integral membrane glycoprotein and homophilic adhesion molecule (Wu et al., 2008) that localizes to the lateral domain of the follicular epithelium (Ng et al., 2016). Ndg, our basal cargo, is a soluble ECM glycoprotein that is secreted in the lower third of the lateral domain and plays a key role in basement membrane assembly and stability in follicle cells (Dai et al., 2018). In attempting to define the membrane domain-specific interactomes of these cargoes, we uncovered how labelling chemistry, radical diffusion and tissue architecture profoundly influence proximity-labelling outcomes, leading to biased and artefactual spatial signatures if not carefully controlled.

## RESULTS

### APEX2–Cad99c is mislocalised to the lateral domain, whereas HRP–Cad99c localizes apically

**Fig. 2.**
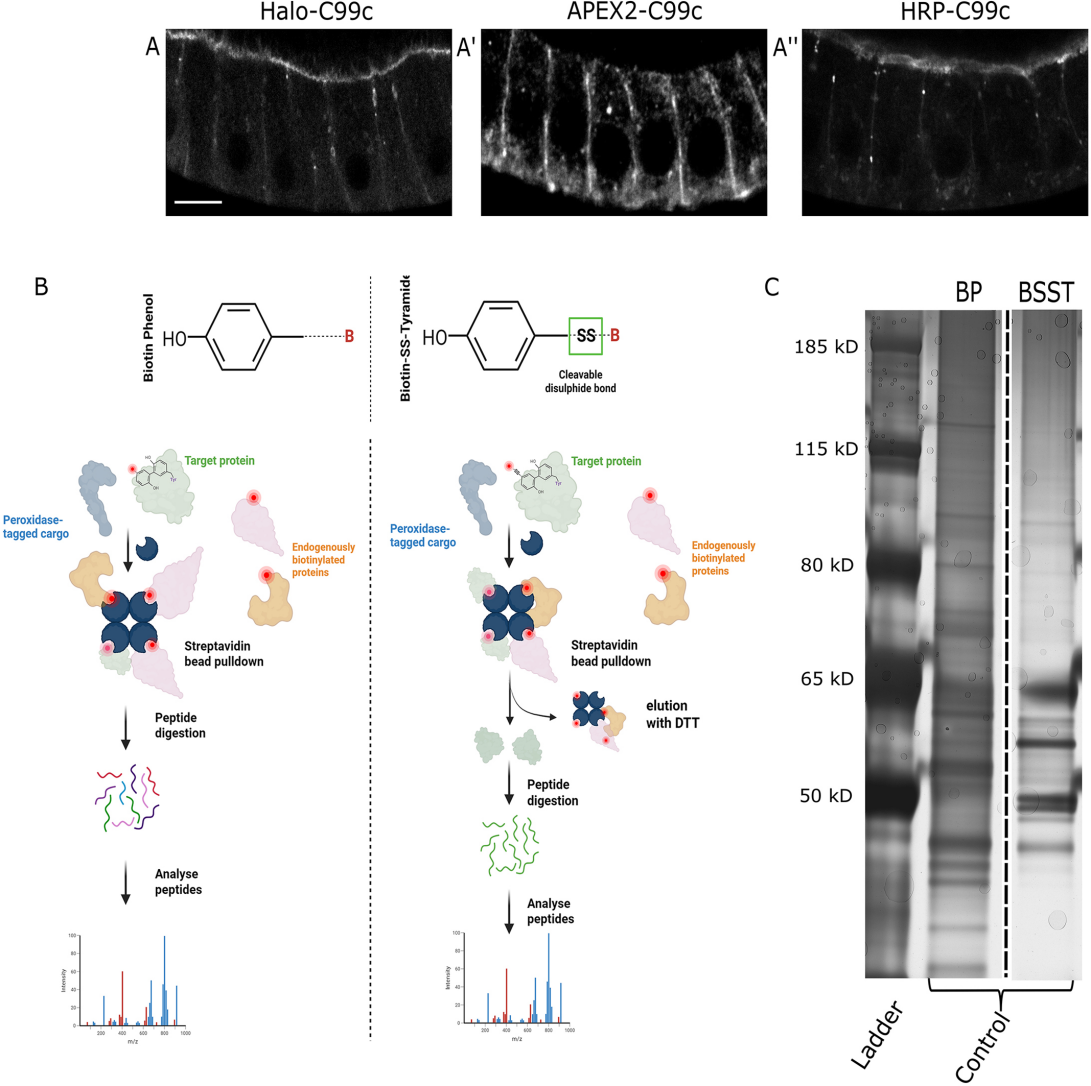
**Proximity labelling with biotin-SS-tyramide reduces background from endogenous biotinylated proteins.** (A–A″) Confocal images comparing the subcellular localization of Cadherin99c (Cad99c or C99c) fused to Halo (A), APEX2 (A′) and HRP (A″) in follicle cells. Halo–Cad99c localized apically, consistent with endogenous Cad99c distribution. In contrast, antibody staining revealed that APEX2–Cad99c mislocalized to the lateral domain, whereas HRP–Cad99c localized predominantly to the apical membrane, similar to the Halo-tagged protein. *n*=3 flies or 6 ovaries stained per cargo. Scale bar: 10 μm. (B) A diagram illustrating peroxidase-mediated proximity labelling using biotin-phenol (BP) or biotin-SS-tyramide (BSST). Both peroxidase-mediated and endogenous biotinylated proteins are enriched during streptavidin pulldown. However, the cleavable disulfide bond in BSST enables selective elution of proximity-labelled proteins with dithiothreitol (DTT), thereby reducing background from endogenous biotinylated proteins, which remain bound to the beads. Created in BioRender by Sen, S., 2026. https://BioRender.com/egh4hco. This figure was sublicensed under CC-BY 4.0 terms. (C) Silver-stained gel showing proteins enriched from control (*yw*) ovaries after labelling with BP or BSST. The BSST lane exhibits a marked reduction in non-specific, high-molecular-mass bands compared with the BP lane, indicating more efficient removal of endogenous biotinylated proteins. *n*=50 ovaries per treatment condition. The dotted line indicates that the image was cropped from a larger gel to juxtapose the two lanes.

To map the membrane proteomes in the follicle cells, first we generated an APEX2–Cad99c fusion construct carrying the signal peptide sequence of Cad99c, APEX2, followed by the rest of Cad99c. APEX2–Cad99c was expressed in the *Drosophila* follicular epithelium using the follicle cell-specific driver *traffic jam-Gal4* (*tj-Gal4*). However, unlike its Halo-tagged version (Halo–Cad99c), which gave an apical signal (Fig. 2A) when labelled with the fluorescent HaloTag ligand, antibody staining against APEX2 indicated a lateral localization for the N-terminally tagged APEX2–Cad99c (Fig. 2A′). Thus, fusion with APEX2 appeared to have overridden the apical trafficking signal of Cad99c, resulting in a different localization pattern from that of the endogenous protein. We therefore decided to use another peroxidase, HRP, in place of APEX2. Antibody staining against HRP showed apical localization for HRP–Cad99c (Fig. 2A″), similar to that for Halo-Cad99c, and we decided to proceed with HRP for the rest of our proximity-labelling experiments. Unlike APEX2, which functions efficiently in intracellular as well as luminal and extracellular environments, HRP is only well-suited for extracellular or luminal applications (Lam et al., 2015). This is because HRP contains four intramolecular disulfide bonds (e.g. Cys41–Cys121, Cys74–Cys79, Cys127–Cys331 and Cys207–Cys239), which are critical for maintaining its structural integrity and enzymatic activity, thereby rendering it unstable in reducing environments, such as the cytosol. APEX2, in contrast, lacks disulphide bonds and is active in all compartments.

### Proximity labelling with biotin-SS-tyramide reduces background signals from endogenous biotinylated proteins

To perform HRP-mediated proximity labelling in the *Drosophila* follicular epithelium, we incubated follicle cells expressing HRP-tagged cargoes with either BP or biotin-SS-tyramide (BSST). The peroxidase catalyses the single-electron oxidation of the biotin label in the presence of H_2_O_2_ to generate a short-lived biotin-phenoxy radical. After 1 min, the reactions were quenched with sodium ascorbate, Trolox and sodium azide. The phenoxy radicals generated by the labelling reaction covalently tag endogenous proteins with biotin in the vicinity of the peroxidase-tagged protein of interest, allowing their subsequent enrichment using streptavidin beads (Fig. 2B) and their identification by mass spectrometry (Hung et al., 2016).

*Drosophila* follicle cells express several high-molecular-mass, endogenous biotinylated proteins (Stevens et al., 2019) that often pose a problem when one is trying to identify proteins that are proximity labelled by HRP-mediated biotinylation via mass spectroscopy. To overcome this problem, we substituted BP with another biotin label, BSST, which has a disulphide bond between the biotin and the phenol moieties (Li et al., 2014) (Fig. 2B). The idea behind this approach is that once the labelled proteins have been pulled down with streptavidin beads, which will purify both the proximity-labelled and endogenous biotinylated proteins, an elution step is performed with a reducing agent such as dithiothreitol (DTT). DTT reduces the disulphide bond between biotin and the proximity-biotinylated proteins, thereby eluting them from the streptavidin beads, while the endogenous biotinylated proteins remain bound. This should reduce the background that is usually seen in such proximity-labelling experiments in follicle cells. This slight change in the labelling technique successfully reduced the background signal in control ovaries that were not expressing HRP-tagged proteins (Fig. 2C). The reduced number of background bands in the lane containing the BBST-treated sample compared to those in the lane containing the BP-treated sample clearly indicates that treatment with BSST is a substantial improvement over the traditional BP-labelling technique (Fig. 2C).

### HRP-tagged Cad99c, Fas3 and Ndg show basal ‘proximity’ labelling

**Fig. 3.**
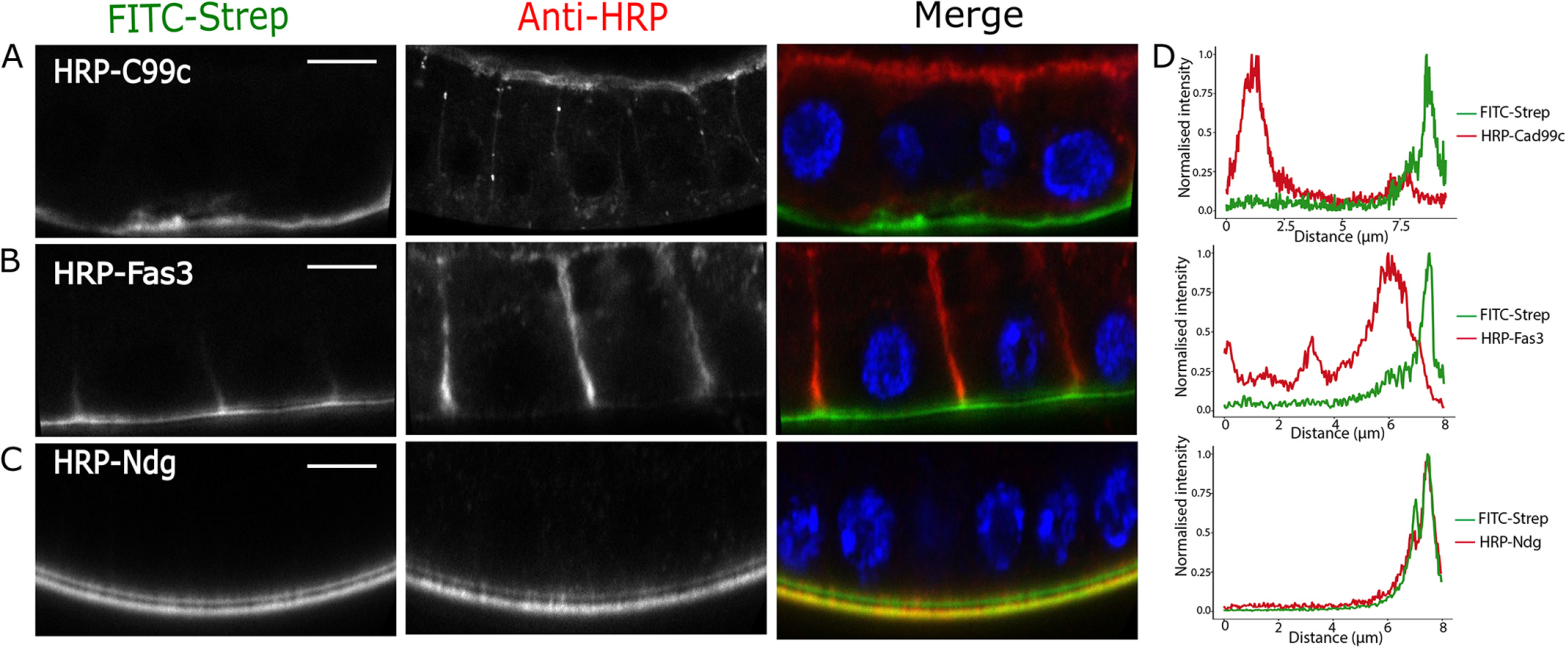
**HRP-mediated proximity labelling of apical and lateral cargoes in follicle cells produces artefactual basal biotinylation.** (A) HRP–Cad99c localized predominantly to the apical membrane, as revealed by anti-HRP staining (red), yet fluorescein–streptavidin (FITC-Strep, green) detected biotinylated proteins almost exclusively at the basal surface after proximity labelling. (B) HRP–Fas3 was confined to the lateral membrane (red), but the biotinylation pattern was also basal (green). (C) In contrast, HRP–Ndg, a soluble basement-membrane cargo, showed colocalization between the anti-HRP signal (red) and FITC-Strep (green) at the basal domain. DAPI (blue) stained the nuclei. *n*=3 flies or 6 ovaries stained per cargo. Scale bars: 10 μm. (D) Quantification of the signal intensity of the anti-HRP staining (red) and FITC-Strep signal (green) as a function of distance from the apical surface.

Antibody staining for the HRP-tagged transmembrane cargoes Cad99c and Fas3 revealed that HRP–Cad99c and HRP–Fas3 localise apically and laterally, respectively, just like their endogenous counterparts (Fig. 3A,B, middle panels), whereas the HRP-tagged soluble cargo Ndg localizes basally (Fig. 3C, middle panel). To visualize the proteins that are proximity labelled for each of our HRP-tagged cargoes, we used fluorescein–streptavidin (FITC-Strep) (Odenwald et al., 2024). As the proximity-labelling technique should only biotinylate proteins within an approximate 20 nm labelling radius of the bait protein (Hung et al., 2014), we hypothesized that the biotinylation pattern observed through FITC-Strep staining should overlap with the antibody staining pattern for each HRP-tagged cargo in the follicular epithelium. Surprisingly, however, the FITC-Strep signal after proximity biotinylation with BSST for all three HRP-tagged proteins was basal (Fig. 3A–D). Almost negligible FITC-Strep labelling was observed in control egg chambers without any BSST treatment (Fig. S1A). Both HRP–Cad99c and HRP–Fas3 showed no or little FITC-Strep signal at the expected position, and only proximity labelling with HRP–Ndg was proximal to the protein of interest (Fig. 3A–C). To quantify the mismatch between the localisation of the bait proteins and the biotinylation signal produced by proximity labelling, we plotted the normalised intensity of the anti-HRP staining for each bait and the corresponding FITC-Strep signal as a function of distance along the apical–basal axis of the epithelium (Fig. 3D). This revealed that the peak of HRP–Cad99c staining was ∼7 μm more apical than the FITC-Strep peak of biotinylation, with the HRP–Fas3 and FITC-Strep peaks being 2 μm apart. Thus, even though our cargoes of interest were localising to their expected destinations, the proteins that were being biotinylated by proximity labelling were very distant from the source of the activated biotin.

### Lowering BSST concentration restores proximity labelling

**Fig. 4.**
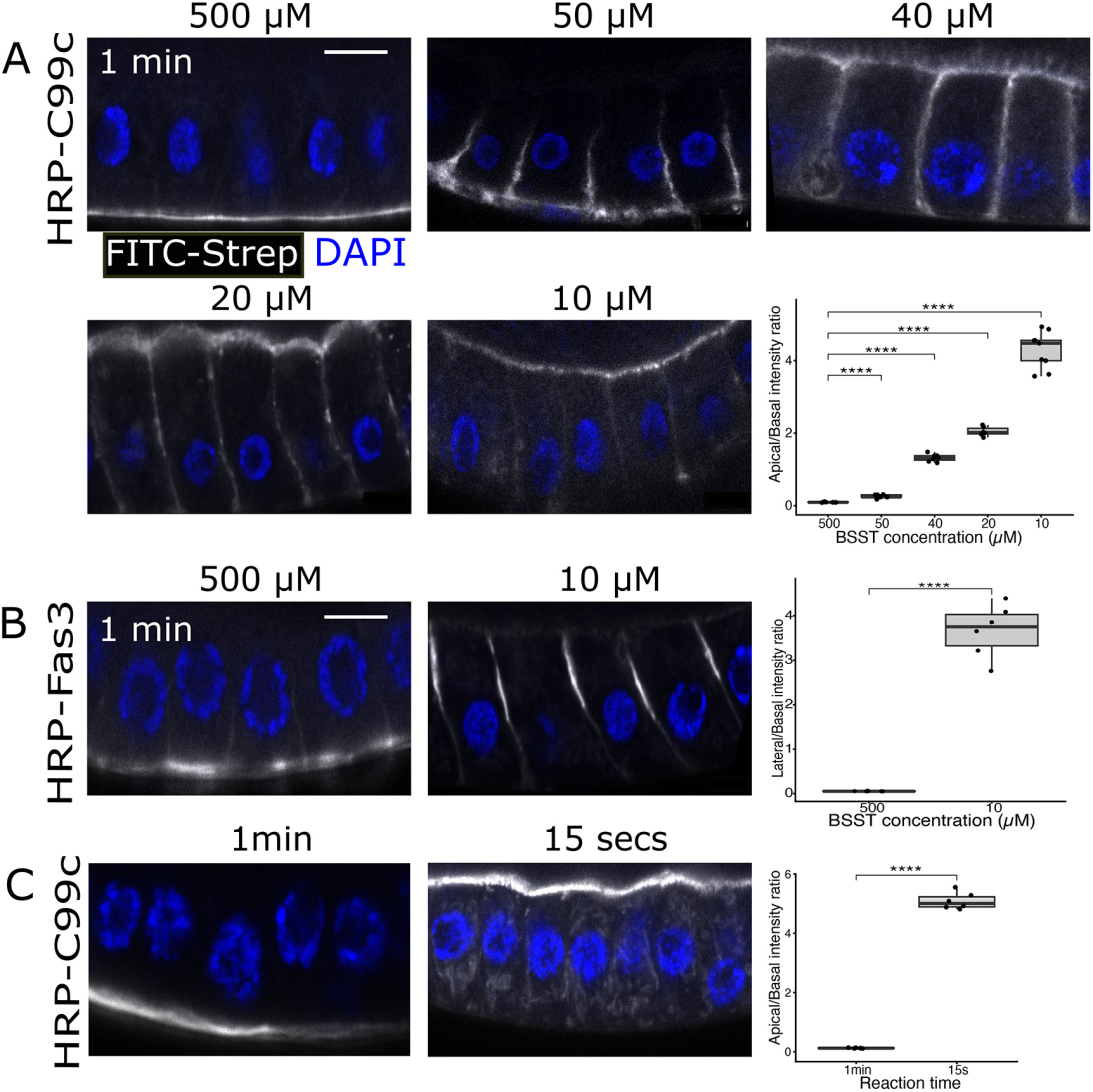
**The range of proximity labelling can be restricted by reducing BSST concentration or reaction time.** (A) Biotinylated proteins visualized by FITC-Strep (white) after proximity labelling with HRP–Cad99c as bait using decreasing concentrations of BSST. At 500 μM BSST, biotinylation was restricted to the basal surface. Progressive dilution of BSST (50 to 10 μM) led to a gradual restoration of apical labelling, with predominantly apical signal observed at 10 μM. (B) The distribution of biotinylated proteins after proximity labelling with HRP–Fas3 as bait showed a similar concentration-dependent behaviour: at 500 μM BSST, the FITC-Strep signal was basal, whereas at 10 μM, it shifted to the lateral membrane, corresponding to the endogenous localization of Fas3. (C) Reducing the labelling duration at constant BSST concentration (500 μM) similarly restored spatial fidelity. After a 1-min reaction, proximity labelling with HRP–Cad99c as bait yielded a basal labelling pattern, whereas shortening the reaction to 15 s resulted in predominantly apical biotinylation. DAPI (blue) stained the nuclei. *n*=3 flies or 6 ovaries stained per condition. Scale bars: 10 μm. The graphs on the right plot the ratio of the FITC-Strep signal intensity at the correct location to the non-proximal basal signal. Data are shown as individual points with box plots indicating the median and interquartile range, and whiskers showing the most extreme values within 1.5× the interquartile range. Statistical significance was determined using unpaired two-tailed Student's *t*-test. ****P<0.0001.

The concentration of the biotin label that is normally used for proximity-labelling reactions with APEX2 or HRP is 500 μM; therefore, we used this concentration for our initial experiments (Lam et al., 2015). When the biotin label BSST was diluted without altering the time of incubation with BSST (30 min) or the labelling reaction with H_2_O_2_ (1 min), the FITC-Strep signals were observed to change from being basal to their expected positions, i.e. apical for Cad99c and lateral for Fas3. At 500 μM BSST, the signal was completely basal for both cargoes (Fig. 4A,B). The FITC-Strep signal became basal and lateral with a 10-fold dilution of BSST (i.e. 50 μM) for HRP–Cad99c; at 40 μM, the signal was more apico–lateral; at 20 μM, the signal became predominantly apical; and HRP–Cad99c finally showed the expected apical localization at 10 μM, as shown by the more than 10-fold increase in the ratio of the intensity of apical FITC-Strepavidin signal to the basal signal (Fig. 4A). Thus, a 50-fold dilution (i.e. 10 μM) of BSST was required to obtain *bona fide* proximity labelling for both the apical and lateral cargoes. These results suggest that the basal signal that we previously observed (Fig. 3A–C) in our proximity-labelling experiments was an artifact caused by the high concentration of the BSST.

Correct proximity labelling for our apical and lateral cargoes was also observed when the time of the labelling reaction was reduced from 1 min to 15 s, keeping the concentration of BSST constant. Under standard conditions, HRP catalyses the production of phenoxy radicals from the biotin label in the presence of H_2_O_2_ for 1 min, before the radicals are quenched. When the time of the reaction was reduced from 1 min to 15 s, the biotinylation pattern changed from being completely basal to the expected apical labelling for HRP–Cad99c, even with higher concentrations (i.e. 500 μM) of BSST (Fig. 4C). Quantification confirmed a significant increase in the apical-to-basal intensity ratio under these conditions (Fig. 4C, right). The observations from Fig. 4A–C led us to conclude that the process of proximity labelling is limited by diffusion. These quantitative measurements were used to define the conditions that restore spatial specificity and were therefore considered optimal for obtaining correct proximity labelling.

By reducing the number of radicals that we generate (through a 50-fold dilution of the biotin label) or by reducing the time for which the radicals are active in the solution (through the addition of the quencher 15 s after addition of H_2_O_2_), we limited the capability of the phenoxy radicals to diffuse from their site of production (proximal to our cargoes of interest) to the basal side of the follicle cells, which seemed to be very rich in binding sites for these electron-deficient phenoxy radicals.

### The basement membrane is a dominant sink for biotin radicals

**Fig. 5.**
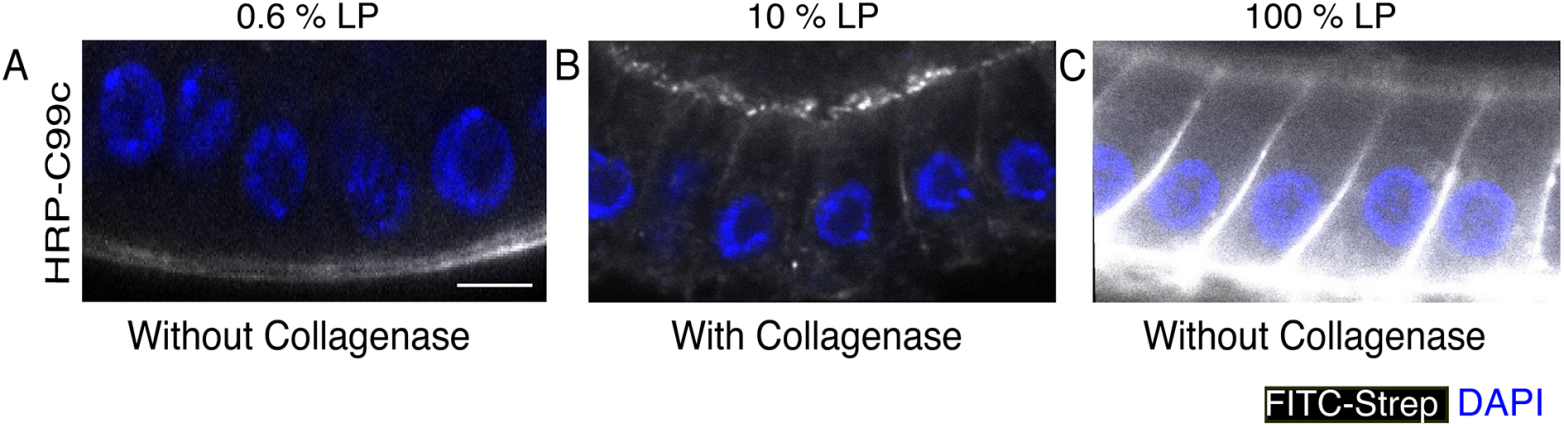
**Basement membrane digestion with collagenase reveals apical proximity labelling with HRP–Cad99c as bait.** (A) Under standard labelling conditions (500 μM BSST, 1 min), FITC-Strep (white) detected a strong basal biotinylation signal at low (0.6%) laser power (LP) in HRP–Cad99c-expressing follicle cells. (B) Following collagenase-mediated digestion of the basement membrane (ECM), the predominant FITC-Strep signal became apical and was readily detectable at comparatively low (10%) laser power, indicating loss of the basal biotinylation sink. (C) In untreated samples, the apical signal was present but obscured by the intense basal signal and became apparent only upon saturation of the basal signal at high (100%) laser power. DAPI (blue) stained the nuclei. *n*=3 flies or 6 ovaries stained per condition. Scale bar: 10 μm.

Our observations so far suggest that the basal side acts as a major sink for diffusing radicals, giving rise to an intense, basal signal. To determine whether it is the basal plasma membrane or the ECM that acts as a sink, we treated the egg chambers with collagenase after proximity labelling. The basal signal was lost after collagenase treatment and a weak apical FITC-Strep signal appeared (Fig. 5B). This apical signal was relatively dim, showing up at approximately 10% laser power, in contrast to the intense, predominantly basal signal under normal conditions (Fig. 5A), which was visible at 0.6% laser power. These results indicate that proximity labelling with HRP–Cad99c does lead to the biotinylation of apical proteins in its vicinity, but these signals are overwhelmed by the basal signal, which is due to the ECM, presumably because the latter provides many reactive sites for the phenoxy radicals. Indeed, a weak apical signal was visible at a 100% laser power in follicle cells treated with the same high concentration of BSST without collagenase treatment (Fig. 5C).

**Fig. 6.**
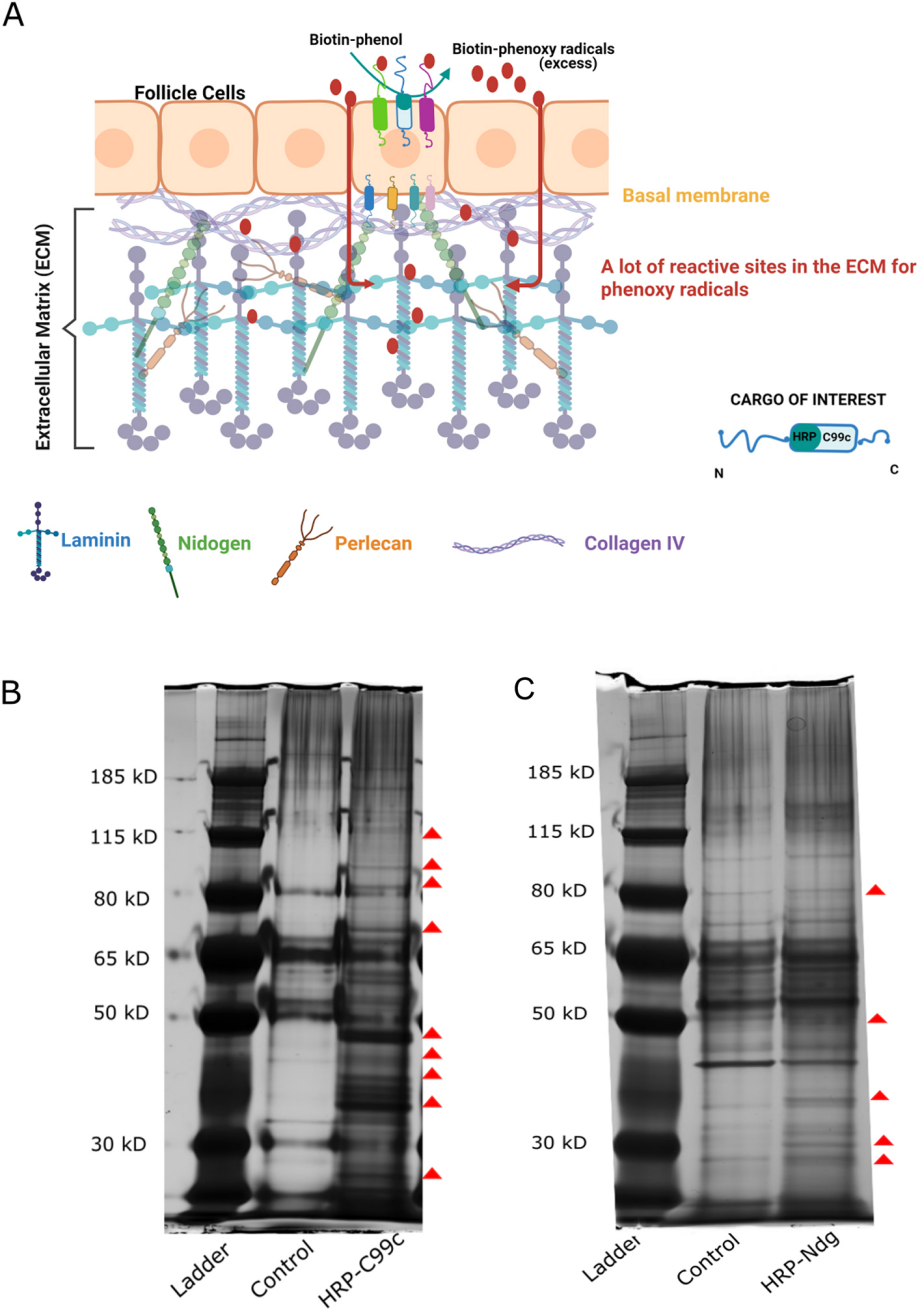
**Phenoxy radical diffusion to the ECM underlies the artefactual basal labelling.** (A) A model illustrating diffusion-controlled proximity labelling using HRP-tagged baits in the *Drosophila* follicular epithelium. Excess biotin label leads to overproduction of long-lived biotin-phenoxy radicals at the apical membrane for HRP–Cad99c, an apical cargo. After saturating proximal binding sites, these radicals diffuse across the cell and are most likely scavenged by the ECM at the basal surface, which is enriched in electron-rich amino acids, functioning as a high-capacity sink for the diffusing radicals. This results in an intense but artefactual basal biotinylation signal. Created in BioRender by Sen, S., 2026. https://BioRender.com/jthuenu. This figure was sublicensed under CC-BY 4.0 terms. (B) Silver-stained gel showing proteins enriched from control (*yw*) ovaries and ovaries expressing HRP–Cad99c after optimized proximity labelling with 40 μM BSST. (C) Silver-stained gel comparing control ovaries with ovaries expressing HRP–Ndg after proximity labelling with 80 μM BSST. *n*=100 ovaries dissected per cargo. Differences in banding patterns between control and HRP-expressing samples are indicated by red arrowheads.

These observations suggest that the ECM contains a large number of binding sites for phenoxy radicals, compared to the extracellular proteins on the other side of the follicle cells, giving rise to an intense basal signal that masks the weaker signals present elsewhere. To ensure that it is not the ECM itself that generates these phenoxy radicals in the absence of any peroxidase, we performed a control experiment in which wild-type ovaries (no peroxidase overexpression) were treated with BSST and H_2_O_2_. No signal was observed with FITC-Strep (Fig. S1B). Thus, the basal biotinylation signal appears, not because the radicals are produced there, but because they diffuse over many thousands of nanometres from their site of production, in sharp contrast to the reported labelling radius of ∼20 nm. The ECM appears to act as a sponge for these radicals and must therefore contain a large number of reactive sites. The ECM components Ndg, Perlecan (also known as Trol), Collagen IVα1 (Col4a1) and α2 (also known as Vkg), and Laminins A (LanA), B1 (LanB1) and B2 (LanB2), and the Dystroglycan (Dg) ECM receptor contain many electron-rich amino acids (such as tyrosine, tryptophan, histidine, cysteine and lysine) that can react with electron-deficient phenoxy radicals, which might explain why they act as a basal sink (Fig. 6A).

Having optimized the concentration of the biotin label (BSST) to produce the expected ‘proximal’ labelling for all three cargoes, we set out to scale up the labelling reactions so that we could identify the labelled proteins. This requires labelling large numbers of whole, undissected ovaries, instead of the dissected egg chambers used for our experiments so far. As this might alter the diffusion of BSST and H_2_O_2_ into the tissue, we re-optimized the BSST concentrations for each cargo. The optimal concentration of BSST was 40 μM for HRP–Cad99c and HRP–Fas3, and 80 μM for HRP–Ndg. We compared the proteins identified by proximity labelling of HRP–Cad99c- and HRP–Ndg-expressing ovaries following this optimised protocol with those from control *yw* ovaries treated identically by running the purified biotinylated proteins after elution with DTT on SDS-PAGE gels, followed by silver staining (Fig. 6B,C). A number of bands appeared only in lanes loaded with the experimental HRP–Cad99c and HRP–Ndg samples, indicating that this protocol can be used to measure the apical and basal proteomes. The differences were more apparent between control and HRP–Cad99c samples than between control and HRP–Ndg samples, suggesting that there are more proteins that can be biotinylated on the apical side than the basal side. The control lanes look slightly different between Fig. 6B and Fig. 6C, possibly because of the concentrations of BSST used for labelling control ovaries for HRP–Cad99c experiments (40 μM) versus HRP–Ndg experiments (80 μM): the higher concentration of BSST might alter the pattern of non-specific labelling within the control ovaries.

### RUSH-mediated intracellular proximity labelling with HRP-tagged cargoes

**Fig. 7.**
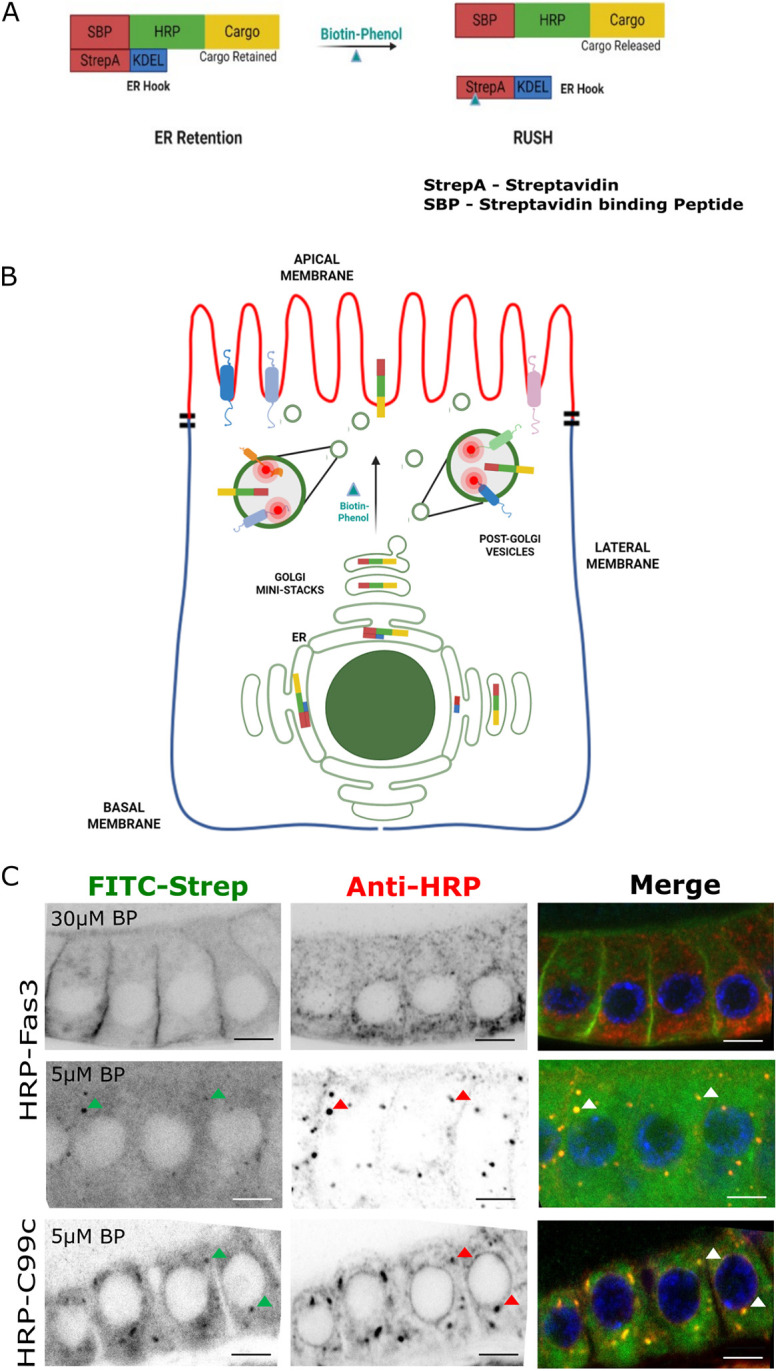
**RUSH-mediated intracellular proximity labelling.** (A) A diagram illustrating the principle of retention using selective hooks (RUSH) combined with proximity labelling. An ER-resident hook (streptavidin–KDEL) retains the reporter protein (SBP–HRP–cargo) in the ER via the SBP–streptavidin interaction. Addition of biotin-phenol (BP) releases the reporter due to the higher affinity of biotin for streptavidin, enabling synchronous trafficking of the HRP-tagged cargo along the secretory pathway. (B) Experimental design for intracellular proximity labelling. After release from the ER, HRP-tagged cargoes transit through the Golgi and post-Golgi vesicles, where proximity labelling is performed to capture compartment-specific interactomes. Created in BioRender by Sen, S., 2026. https://BioRender.com/pq46mel. This figure was sublicensed under CC-BY 4.0 terms. (C) Following 15 min of RUSH with 30 μM BP, HRP–Fas3 localized to Golgi and post-Golgi vesicles as shown by anti-HRP staining (red puncta), whereas FITC-Strep (green) detected predominant biotinylation at the lateral membrane. At a lower BP concentration (5 μM), FITC-Strep signal (green arrowheads) colocalized with anti-HRP staining (red arrowheads) for HRP–Fas3 and HRP–Cad99c in Golgi and post-Golgi compartments, yielding spatially faithful intracellular proximity labelling. DAPI (blue) stained the nuclei. *n*=3 flies or 6 ovaries stained per cargo/per condition. Scale bars: 10 μm.

We combined the retention using selective hooks (RUSH) system with the proximity-labelling protocol to perform proximity labelling for our apical and lateral cargoes (HRP–Cad99c and HRP–Fas3) at the Golgi and in post-Golgi vesicles. RUSH involves the expression of two fusion proteins: (1) a hook – streptavidin fused to the endoplasmic reticulum (ER) retention signal KDEL, which is stably retained in the ER, and (2) a reporter protein containing the streptavidin-binding peptide (SBP), which interacts with the hook until it is outcompeted by the addition of biotin (Boncompain et al., 2012). Upon adding biotin, the reporter is released from the hook, producing a synchronous wave of cargo that traffics through the secretory pathway (Fig. 7A,B).

The SBP sequence was added into the HRP-tagged constructs after the signal peptide sequence. Follicle cells expressing the hook and SBP–HRP-tagged cargoes were treated with H_2_O_2_ in the presence of biotin label (BP) at specific time points after the release of the cargo from the ER. This should label nearby proteins when the cargo reaches its peak concentration in the compartment of interest, such as the the Golgi, post-Golgi vesicles or recycling endosomes. Following labelling, the cells were stained with FITC-Strep to reveal the presence of biotinylated proteins, which should be potential interaction partners of our cargoes of interest in different compartments of the secretory pathway. Here, we used BP instead of BSST for intracellular labelling as the reducing environment of the cytosol would otherwise reduce the disulphide bond in BSST, making biotinylation impossible.

We were interested in labelling the interactome of our cargoes of interest (HRP–Cad99c and HRP–Fas3) in the Golgi and post-Golgi compartments. We therefore released the SBP–HRP-tagged cargoes with BP, which induces release from the hook as efficiently as biotin alone. We then carried out the proximity labelling with H_2_O_2_ for 1 min, 15 min after the addition of BP, when the cargoes reach their peak in Golgi and post-Golgi compartments (Richens et al., 2025). The proximity labelling for HRP–Fas3 in the presence of 30 μM BP at the 15 min time point appeared on the membrane, as visualized with FITC-Strep staining (Fig. 7C), whereas antibody staining indicated the expected protein localization in Golgi and post-Golgi compartments. When the concentration of the BP was reduced to 5 μM, the proximity labelling now coincided with the expected protein localization in the Golgi and post-Golgi compartments for both HRP–Fas3 and HRP–Cad99c (Fig. 7C). This is consistent with our earlier observations regarding diffusion-controlled proximity labelling of our HRP-tagged cargoes at steady state. In this case, the plasma membrane appears to have more reactive sites (i.e. electron-rich amino acids) for the phenoxy radicals than the Golgi or post-Golgi compartments. The wave of secreted proteins after RUSH was not entirely synchronous, and some cargo had already reached the plasma membrane by 15 min. When the biotin-phenoxy radicals are produced in excess, they will produce stronger labelling in regions with the greatest number of targets, causing intense plasma membrane signals that mask the labelling of internal compartments. Once the concentration of the radicals was reduced, the observed that the signal coincided with the localization patterns of the HRP-tagged proteins. This highlights the importance of titrating the level of biotin-phenoxy radicals produced to match the local density of reactive target proteins.

## DISCUSSION

While attempting to carry out proximity labelling in *Drosophila* follicle cells using HRP-tagged cargoes following the protocol in the literature, we realized that proximity labelling is a diffusion-controlled process. Using the concentration of the biotin label (BSST) recommended in the protocol, we obtained basal biotinylation patterns for our apical (HRP–Cad99c), lateral (HRP–Fas3) and basal (HRP–Ndg) cargoes, despite antibody staining confirming their expected localization. The proximity labelling therefore appeared to be very distal to our cargoes of interest. When we reduced the number of phenoxy radicals generated by lowering the concentration of the biotin label or decreasing the reaction time, the artefactual basal signal disappeared and we observed proximity labelling in the vicinity of the HRP-tagged proteins.

We believe that this long-distance labelling observed at high BP or BSST concentrations is due to the relatively long half-life (milliseconds) of the phenoxy radicals generated during the proximity-labelling reaction (Oakley et al., 2022). This has been attributed to the resonance stabilization of the radical intermediate, in which the unpaired electron is delocalized across the aromatic ring and oxygen atom, lowering its reactivity and prolonging its persistence in biological environments (Coppinger, 1957; Dellinger et al., 2007; Wiater et al., 2000) For BP-based peroxidase labelling using stimulated emission depletion microscopy, Oakley et al. (2022) reported that individual proximity-labelling clusters had a full width at half maximum (FWHM) of 269±41 nm in contrast to the previously reported 20 nm (Martell et al., 2012). However, our data pointed towards even longer diffusion distances, ranging from about 5 to 8 μm.

The distances over which phenoxy radicals can diffuse can be estimated from classical diffusion theory. The root mean square displacement of a diffusing molecule is given by ⟨x^2^⟩=2Dt (in one dimension) or 6Dt (in three dimensions), where D is the diffusion coefficient and t is the lifetime of the species. Using the estimated diffusion coefficient for BP radicals of 200 μm s^−1^ (Yang et al., 2025) and lifetimes in the low millisecond range, this yields diffusion distances in the range of 0.6 to 1.0 μm, which is much larger than the 20–300 nm reported by Rhee et al. (2013) and Oakley et al. (2022), but significantly smaller than the distance from the apical membrane to the basal membrane of the follicle cells, which is 5–8 μm. This suggests that the half-life of phenoxy radicals depends on their probability of reacting with an electron-rich target and, once the local targets are saturated, they can continue diffusing for more than 10 ms until they react. It is therefore very important to titrate the concentration of the radicals based on the local density of available reactive sites, particularly for biotin-phenoxy-mediated proximity-labelling approaches.

Phenoxy radical-based proximity-labelling methods such as APEX2 and HRP generate radicals with lifetimes that allow diffusion over distances of several micrometres, unless diffusion is limited by a barrier, such as the double membranes that surround mitochondria, or by a high local density of reactive target proteins (Lam et al., 2015). Our results therefore highlight the importance of titrating radical production to match the local density of reactive targets. Based on these findings, we propose several practical guidelines for peroxidase-based proximity labelling in epithelial tissues. First, BP or BSST concentrations should be empirically titrated, as standard concentrations (e.g. ∼500 μM) can lead to extensive diffusion and loss of spatial specificity. In our system, concentrations in a much lower range (10–40 μM) were sufficient to restore domain-restricted labelling while maintaining detectable signal. Second, reducing the duration of the labelling reaction can similarly improve spatial resolution; shortening reaction times (e.g. from 1 min to 15 s) significantly limited diffusion. Third, optimisation should be guided by image-based validation, ensuring that the biotinylation signal matches the known localization of the bait protein. Finally, tissues with abundant ECMs or high densities of reactive residues might require more stringent control of radical production, as these environments can act as high-capacity sinks for diffusing radicals. Together, these considerations provide a practical framework for optimizing proximity-labelling conditions to achieve spatially faithful proteomic mapping.

The problem of extended diffusion distances can be solved by proximity labelling with shorter-lived radical species that cannot diffuse so far. A recent technique called μMap employs photocatalysts (e.g. an iridium complex) that convert diazirine-biotin into extremely short-lived carbene species (<1 ns) upon blue-light irradiation, reacting almost instantly within a sub-nanometre radius before quenching (Geri et al., 2020). This method yields highly localized labelling, thereby offering superior temporal and spatial control. For high-precision, minimal-diffusion labelling, carbene-based approaches such as μMap currently offer the best solution compared to phenoxy-based systems and are possibly ideal for mapping protein microenvironments in extracellular spaces (Bartholow et al., 2024; Ng et al., 2025). Nitrene-based (aryl azide photolysis) and singlet oxygen-based proximity-labelling approaches, such as mini-Singlet Oxygen Generator (miniSOG) and Singlet Oxygen Photosensitizing Protein (SOPP), generate reactive singlet oxygen (^1^O_2_) upon light activation, enabling protein labelling through photo-oxidative chemistry. These methods provide additional photochemical flexibility but have moderate diffusion-related limitations and potential phototoxicity concerns (Westberg et al., 2017; Tay et al., 2023).

Multiple model systems are increasingly being interrogated using proximity-labelling-based proteomics. Some examples include use of APEX2 to enable tissue- and subcellular-specific identification and quantification of proteins in *Caenorhabditis elegans* (Reinke et al., 2017), TurboID profiling of protein complexes in mammalian epithelial cells, worms and fly wing discs (Branon et al., 2018), and tissue-specific proteomic profiling in zebrafish (Xiong et al., 2021).

These approaches rely on fundamentally distinct labelling chemistries. Peroxidase-based methods such as HRP and APEX2 generate diffusible phenoxy radicals, which can travel considerable distances before reacting with target proteins. As a result, the effective labelling radius is strongly influenced by radical lifetime, local target density and diffusion constraints, as demonstrated in this study. It has recently been proposed that the biotin-AMP intermediate produced by engineered biotin ligases, such as BioID and TurboID, remains associated with the biotin ligase for up to 10 s and is transferred to lysine residues on nearby proteins through direct contact, without the involvement of a freely diffusible radical species (Yang et al., 2025). Thus, biotin ligase-based approaches might be more spatially constrained and less susceptible to diffusion-driven artefacts, although they are limited by slower kinetics and a requirement for sustained protein proximity.

These mechanistic differences highlight that the choice of proximity-labelling strategy should be guided by the specific biological context and the desired balance between temporal resolution, spatial precision and labelling efficiency. Collectively, studies across multiple systems demonstrate that proximity labelling combined with mass spectrometry can reveal compartment-specific interactomes in a wide range of polarized tissues, while also underscoring the importance of tailoring labelling conditions to each experimental context.

In this work, we have developed and applied proximity labelling to the different membrane domains in polarized follicle cells. Through systematic optimization, this work revealed that it is crucial to match the concentration of biotin radicals produced to the number of available reactive sites near the bait. These findings provide a practical framework for improving spatial accuracy in peroxidase-based proximity labelling and establish a stronger technical foundation for future proteomic studies in *Drosophila* and other systems in which radical diffusion might influence labelling outcomes.

## MATERIALS AND METHODS

### Fly husbandry and lines

Fly stocks employed in this study are listed in Table S1.

For non-RUSH-based imaging and proteomics purposes, flies were raised at 25°C. For RUSH experiments, flies were raised at 25°C until pupation, at which point they were moved to 18°C to reduce leakiness of the RUSH system. If kept at 25°C for the entire duration, due to the imbalance between the expression levels of the ER hook and the cargo, most of the cargo was released and localized at the plasma membrane before the addition of biotin. Shifting to 18°C restored the balance in the expression levels of the hook and the cargo and minimized the number of cargoes in the non-bound state.

All primers used for making the transgenic flies were designed using the NEBuilder HiFi assembly protocol (New England Biolabs, E5520S) and their sequences are provided in Table S2.

UAST-SBP-APEX2-Cad99c was created by cloning the sequence encoding the signal peptide of Cad99c followed by a GSGSGSG linker, the sequence for SBP, a Pro–Ala–Gly linker, then the APEX2 sequence, another GSGSGSG linker, and the rest of Cad99c into the pUAST-attb vector (DGRC Stock 1419; https://dgrc.bio.indiana.edu//stock/1419; RRID:DGRC_1419) using NEBuilder HiFi assembly. UAST-SBP-HRP-Cad99c and UAST-SBP-HRP-Fas3 were created by cloning the sequence encoding the signal peptide of Cad99c or Fas3 followed by a GSGSGSG linker, the sequence for SBP, a Pro–Ala–Gly linker, then the HRP sequence, another GSGSGSG linker, and the rest of Cad99c or Fas3 into the same vector using NEBuilder HiFi assembly. UAST-SBP-HRP-Ndg was created by cloning the sequencing encoding the signal peptide of cg25c (sequence of *Drosophila* Col4a1) followed by the SBP sequence, then a Pro–Ala–Gly linker, then the HRP sequence, a GSHMRSRPTS linker and the Ndg sequence into the pUAST-attb vector using NEBuilder HiFi assembly.

The primers used for making these constructs are numbered 1–6 for APEX2–Cad99c in the primer details table (Table S2). The APEX2 sequence was cloned from pcDNA3 Connexin43-GFP-APEX2 (Addgene plasmid 49385) and the HRP sequence from pCT2-HRP (Addgene plasmid 44021). The primers used for making the constructs HRP–Cad99c are numbered 1, 6 and 7–10; the primers used to make HRP–Fas3 are 11–16; and those for HRP–Ndg are 17–22.

The cargoes were designed to ensure that SBP was facing the lumen of the ER and therefore could access the streptavidin-KDEL hook. Transgenic flies were made by injecting into the attp2 landing site as mentioned in Table S1 (Groth et al., 2004). The genotype for the attp2 site used here was Py[+t7.7]=nanos-phiC31-nt.NLSX;Py[+t7.7]=CaryPattP2 and the recombinase used was ΦC31 integrase.

### Proximity labelling in different *Drosophila* epithelial tissues

Flies were fattened on yeast for 1 day at 25°C. Ovarioles were dissected out of ovaries and the muscle sheath in Schneider's insect medium (Sigma-Aldrich, S0146) supplemented with insulin (7.5 μg/ml, Merck, I9278). The ovarioles were then incubated in different concentrations of BSST (Iris Biotech, LS-3570) or BP (Merck, 41994-02-9) labelling buffer for 30 min at room temperature (RT). Freshly made 100 mM H_2_O_2_ was then added to the labelling buffer to achieve a final concentration of 1 mM and the samples were incubated for 1 min. The samples were then quenched by addition of freshly prepared quencher solution three times [1 M sodium ascorbate, 500 mM Trolox (Merck, 648471) and 1 M sodium azide] three times (Hung et al., 2016), followed by fixation in 4% formaldehyde in PBS containing 0.2% Tween 20 (PBT) for 20 min with rotation. The samples were then washed three times for 10 min in PBT. To view the proximity-labelled proteins, egg chambers were then incubated with FITC-Strep (Thermo Fisher Scientific, S-869, 1:500) in PBT for 1 h at RT, washed three times for 10 min in PBT and then mounted in Vectashield (Vecta Labs) for imaging.

### Optimisation of BSST concentration

To determine optimal labelling conditions, the concentration of BSST and reaction time were systematically varied. Optimal conditions were defined based on a combination of spatial specificity, signal intensity and reproducibility. Spatial specificity was assessed by comparing the distribution of the FITC-Strep signal with the expected localization of the HRP-tagged cargoes. This was quantified by measuring the ratio of fluorescence intensity in the correct membrane domain (apical for HRP–Cad99c, lateral for HRP–Fas3) relative to the basal domain as described.

Conditions were considered optimal when they produced a high signal in the correct domain relative to the basal domain (correct domain-to-basal intensity ratio) while maintaining sufficient signal intensity in the expected domain for reliable detection and consistent results across independent samples. Final concentrations used for proteomic experiments were selected based on these criteria.

### Collagenase treatment of egg chambers

Following fixation, proximity-labelled egg chambers were treated with 0.05% collagenase type I (Merck, SCR103) in a Ca^2+^-containing collagenase buffer (100 mM Tris pH 8.0, 500 mM NaCl and 40 mM CaCl_2_) at RT for 30 min (Töpfer et al., 2022).

### Halo staining of fixed egg chambers

For imaging of egg chambers expressing Halo–Cad99c, ovarioles were dissected in Schneider's insect medium as described above and fixed in 4% formaldehyde in PBS for 20 min with rotation. After fixation, samples were washed three times for 5 min in PBS, then incubated with Halo-TMR ligand (Promega, G8252) for 1 h at 37°C with shaking at 500 rpm (Richens et al., 2025). Samples were washed six times for 10 min in PBS containing 0.1% Triton X-100 (PBX) and then mounted for imaging.

### Antibody staining of egg chambers

For antibody staining of fixed RUSH samples, RUSH was carried out in egg chambers expressing SBP–HRP–Cad99c or SBP–HRP–Fas3. Egg chambers were dissected in Schneider's insect medium supplemented with insulin and incubated in 400 μM biotin for 15 min (to enable RUSH to proceed to the trans-Golgi and post-Golgi vesicles). For imaging of egg chambers expressing APEX2- or HRP-tagged cargoes under steady-state conditions, ovaries of fattened flies were dissected into Schneider's medium as usual but without biotin. Ovarioles were then transferred to 4% formaldehyde in PBT and fixed for 15 min with rotation. Ovarioles were washed three times for 10 min in PBT, incubated with Alexa Fluor 647 Phalloidin (1:500, Thermo Fisher Scientific, A22287) in PBT for actin staining for 30 min at RT, washed in PBT once for 10 min and then blocked in PBT with 10% BSA for 1 h. Ovarioles were then incubated overnight at 4°C in PBT containing 1% BSA with the primary antibody. Ovarioles were washed four times for 30 min in PBT containing 1%, BSA, then stained for 4 h at RT with 1:500 secondary antibody in PBT containing 0.1% BSA. Samples were washed three times for 10 min in PBT and mounted in Vectashield plus DAPI (Vector Labs) (Richens et al., 2025). Images were acquired on a Leica SP8 WLL confocal microscope with a 63× oil objective r 1.4 NA.

The primary antibodies used in this study were rabbit anti-APEX2 (LSBio, C285734, 1:1000) and Alexa Fluor 647-fused goat anti-HRP (Jackson Immunoresearch, AB-2338967, 1:500). The secondary antibodies used in this study include goat anti-rabbit Alexa Fluor 647 (Thermo Fisher Scientific, A-21245; 1:500).

### Image analysis

Confocal images were analysed using Fiji/ImageJ. Fluorescence intensity profiles were measured in single apical–basal sections through the midplane of follicle cells (Fig. 3D) using a straight-line region of interest (ROI) (5–10 pixels in width) drawn perpendicular to the epithelial surface. The same ROI was applied to both the anti-HRP and FITC-Strep channels. Raw fluorescence intensities were exported and analysed in Rstudio. Background intensity, measured from regions outside the tissue, was subtracted from each channel. Intensity profiles were normalized to the maximum value within each channel to enable comparison of spatial distributions. The position of peak intensity for each channel was determined as the distance corresponding to the maximum value of the intensity profile. The displacement between the HRP signal and the biotinylation signal was calculated as the difference between the peak positions along the apical–basal axis. Multiple cells from independent egg chambers were analysed for each condition (*n*=15 cells per cargo), and representative profiles are shown as indicated in the figure legends.

The spatial redistribution of the biotinylation signal in Fig. 4 was quantified using region-based measurements. Apical (or lateral) and basal ROIs were defined based on cell morphology, with the apical ROI positioned at the apical (for HRP–Cad99c) or lateral (for HRP–Fas3) membrane, and the basal ROI encompassing the basal surface and adjacent ECM. The mean fluorescence intensity of the FITC-Strep signal was measured within each ROI using Fiji (*n*=8–10 cells per condition). The background intensity was subtracted as described above. The apical-to-basal (or lateral-to-basal) intensity ratio was calculated for each cell and used for statistical analysis. Data were analysed in Rstudio and plotted as box plots with individual data points. Statistical comparisons were performed as indicated in the figure legends.

### Membrane fraction isolation from *Drosophila* ovaries for silver staining gels

Flies were fattened on yeast for 1 day at 25°C. Splayed-out ovaries were dissected out of the flies in Schneider's insect medium supplemented with insulin as described above. The ovaries were then proximity labelled by BSST as mentioned previously and then homogenized in IP lysis buffer (Thermo Fisher Scientific, 87787) supplemented with 1× phosphatase inhibitors (Thermo Fisher Scientific,78420) and 1× protease inhibitor (Thermo Fisher Scientific, A32965) in 1.5 ml Eppendorf tubes on ice. The tissue lysate was then centrifuged at 720 ***g*** for 5 min at 4°C. The pellet containing the nuclei was discarded and the supernatant containing the cytoplasm, membrane and mitochondria was retained.

The supernatant was transferred into a fresh tube and kept on ice. It was then centrifuged at 10,000 ***g*** for 5 min at 4°C. The pellet obtained contained mitochondria. The supernatant was then transferred to a fresh tube and kept on ice. This contained the cytoplasm and the membrane fraction, which was eventually used for the pulldown. Streptavidin-coated magnetic beads (New England Biolabs, S1420S) were blocked with lysis buffer containing 1% BSA for 1 h at RT with rotation, followed by two quick washes with lysis buffer. The tissue extract containing the cytoplasm and the membrane fraction was incubated with the magnetic beads with rotation for 1 h at RT in the presence of 200 mM NaCl. After binding, the solution was removed and the beads were dissolved in lysis buffer and transferred to a fresh tube. The beads were then washed with lysis buffer, 1 M KCl, 0.1 M Na_2_CO_3_, 2 M urea in 10 mM Tris HCl, pH 8, and then again with lysis buffer for 10 min each (Hung et al., 2016). After the washes, the beads were transferred to another fresh tube and eluted with 10 mM DTT for 25 min at RT, followed by alkylation with 50 mM iodoacetamide for 10 min in the dark.

The eluant was then collected and mixed with LDS sample buffer (Thermo Fisher Scientific, B0007) and run on a NuPage 8% BisTris gel (Thermo Fisher Scientific, NW00080BOX), followed by silver staining to visualize the protein bands (Thermo Fisher Scientific, 24612).

## Supplementary Material



10.1242/joces.264887_sup1 Supplementary information

## References

[JCS264887C1] Bartholow, T. G., Burroughs, P. W. W., Elledge, S. K., Byrnes, J. R., Kirkemo, L. L., Garda, V., Leung, K. K. and Wells, J. A. (2024). Photoproximity labeling from single catalyst sites allows calibration and increased resolution for carbene labeling of protein partners in vitro and on cells. *ACS Central Sci.* 10, 199-208. 10.1021/acscentsci.3c01473 PMC10823516 38292613

[JCS264887C2] Boncompain, G., Divoux, S., Gareil, N., De Forges, H., Lescure, A., Latreche, L., Mercanti, V., Jollivet, F., Raposo, G. and Perez, F. (2012). Synchronization of secretory protein traffic in populations of cells. *Nat. Methods* 9, 493-498. 10.1038/nmeth.1928 22406856

[JCS264887C3] Branon, T. C., Bosch, J. A., Sanchez, A. D., Udeshi, N. D., Svinkina, T., Carr, S. A., Feldman, J. L., Perrimon, N. and Ting, A. Y. (2018). Efficient proximity labeling in living cells and organisms with TurboID. *Nat. Biotechnol.* 36, 880-887. 10.1038/nbt.4201 30125270 PMC6126969

[JCS264887C4] Coppinger, G. M. (1957). A stable phenoxy radical inert to Oxygen. *J. Am. Chem. Soc.* 79, 501-502. 10.1021/ja01559a073

[JCS264887C5] Dai, J., Estrada, B., Jacobs, S., Sánchez-Sánchez, B. J., Tang, J., Ma, M., Magadán-Corpas, P., Pastor-Pareja, J. C. and Martín-Bermudo, M. D. (2018). Dissection of Nidogen function in Drosophila reveals tissue-specific mechanisms of basement membrane assembly. *PLoS Genet.* 14, e1007483. 10.1371/journal.pgen.1007483 30260959 PMC6177204

[JCS264887C6] Dellinger, B., Lomnicki, S., Khachatryan, L., Maskos, Z., Hall, R. W., Adounkpe, J., McFerrin, C. and Truong, H. (2007). Formation and stabilization of persistent free radicals. *Proc. Combust. Inst.* 31, 521-528. 10.1016/j.proci.2006.07.172 25598747 PMC4295210

[JCS264887C7] Geri, J. B., Oakley, J. V., Reyes-Robles, T., Wang, T., McCarver, S. J., White, C. H., Rodriguez-Rivera, F. P., Parker, D. L., Jr, Hett, E. C., Fadeyi, O. O. et al. (2020). Microenvironment mapping via Dexter energy transfer on immune cells. *Science* 367, 1091-1097. 10.1126/science.aay4106 32139536 PMC7336666

[JCS264887C8] Groth, A. C., Fish, M., Nusse, R. and Calos, M. P. (2004). Construction of transgenic Drosophila by using the site-specific Integrase from phage ϕC31. *Genetics* 166, 1775-1782. 10.1093/genetics/166.4.1775 15126397 PMC1470814

[JCS264887C9] Guo, J., Guo, S., Lu, S., Gong, J., Wang, L., Ding, L., Chen, Q. and Liu, W. (2023). The development of proximity labeling technology and its applications in mammals, plants, and microorganisms. *Cell Commun. Signal.* 21, 269. 10.1186/s12964-023-01310-1 37777761 PMC10544124

[JCS264887C10] Gurudev, N., Yuan, M. and Knust, E. (2014). Chaoptin, prominin, eyes shut and crumbs form a genetic network controlling the apical compartment of *Drosophila* photoreceptor cells. *Biol. Open* 3, 332-341. 10.1242/bio.20147310 24705015 PMC4021355

[JCS264887C11] Hung, V., Zou, P., Rhee, H.-W., Udeshi, N. D., Cracan, V., Svinkina, T., Carr, S. A., Mootha, V. K. and Ting, A. Y. (2014). Proteomic mapping of the human mitochondrial intermembrane space in live cells via ratiometric APEX tagging. *Mol. Cell* 55, 332-341. 10.1016/j.molcel.2014.06.003 25002142 PMC4743503

[JCS264887C12] Hung, V., Udeshi, N. D., Lam, S. S., Loh, K. H., Cox, K. J., Pedram, K., Carr, S. A. and Ting, A. Y. (2016). Spatially resolved proteomic mapping in living cells with the engineered peroxidase APEX2. *Nat. Protoc.* 11, 456-475. 10.1038/nprot.2016.018 26866790 PMC4863649

[JCS264887C13] Isabella, A. J. and Horne-Badovinac, S. (2016). Rab10-mediated secretion synergizes with tissue movement to build a polarized basement membrane architecture for organ morphogenesis. *Dev. Cell* 38, 47-60. 10.1016/j.devcel.2016.06.009 27404358 PMC4942852

[JCS264887C14] Laffafian, A. and Tepass, U. (2019). Identification of genes required for apical protein trafficking in Drosophila photoreceptor cells. *G3 (Bethesda)* 9, 4007-4017. 10.1534/g3.119.400635 31649044 PMC6893196

[JCS264887C15] Lam, S. S., Martell, J. D., Kamer, K. J., Deerinck, T. J., Ellisman, M. H., Mootha, V. K. and Ting, A. Y. (2015). Directed evolution of APEX2 for electron microscopy and proximity labeling. *Nat. Methods* 12, 51-54. 10.1038/nmeth.3179 25419960 PMC4296904

[JCS264887C16] Li, X.-W., Rees, J. S., Xue, P., Zhang, H., Hamaia, S. W., Sanderson, B., Funk, P. E., Farndale, R. W., Lilley, K. S., Perrett, S. et al. (2014). New insights into the DT40 B cell receptor cluster using a proteomic proximity labeling assay. *J. Biol. Chem.* 289, 14434-14447. 10.1074/jbc.M113.529578 24706754 PMC4031500

[JCS264887C17] Liu, Q., Zheng, J., Sun, W., Huo, Y., Zhang, L., Hao, P., Wang, H. and Zhuang, M. (2018). A proximity-tagging system to identify membrane protein–protein interactions. *Nat. Methods* 15, 715-722. 10.1038/s41592-018-0100-5 30104635

[JCS264887C18] Martell, J. D., Deerinck, T. J., Sancak, Y., Poulos, T. L., Mootha, V. K., Sosinsky, G. E., Ellisman, M. H. and Ting, A. Y. (2012). Engineered ascorbate peroxidase as a genetically encoded reporter for electron microscopy. *Nat. Biotechnol.* 30, 1143-1148. 10.1038/nbt.2375 23086203 PMC3699407

[JCS264887C19] Ng, B. F., Selvaraj, G. K., Mateos, C. S.-C., Grosheva, I., Alvarez-Garcia, I., Martín-Bermudo, M. D. and Palacios, I. M. (2016). α-spectrin and integrins act together to regulate actomyosin and columnarization, and to maintain a monolayered follicular epithelium. *Development* 143, 1388-1399. 10.1242/dev.130070 26952981 PMC4852512

[JCS264887C20] Ng, H. K., Douglas, C. J. and Seath, C. P. (2025). μMap Photoproximity labeling on the cell surface. *Curr. Protoc.* 5, e70216. 10.1002/cpz1.70216 41060711 PMC12507076

[JCS264887C21] Oakley, J. V., Buksh, B. F., Fernández, D. F., Oblinsky, D. G., Seath, C. P., Geri, J. B., Scholes, G. D. and Macmillan, D. W. C. (2022). Radius measurement via super-resolution microscopy enables the development of a variable radii proximity labeling platform. *Proc. Natl. Acad. Sci. USA* 119, e2203027119. 10.1073/pnas.2203027119 35914173 PMC9371666

[JCS264887C22] Odenwald, J., Gabiatti, B., Braune, S., Shen, S., Zoltner, M. and Kramer, S. (2024). Detection of TurboID fusion proteins by fluorescent streptavidin outcompetes antibody signals and visualises targets not accessible to antibodies. *eLife* 13, RP95028. 10.7554/elife.95028 39206942 PMC11361705

[JCS264887C23] Reinke, A. W., Mak, R., Troemel, E. R. and Bennett, E. J. (2017). In vivo mapping of tissue- and subcellular-specific proteomes in Caenorhabditis elegans. *Sci. Adv.* 3, e1602426. 10.1126/sciadv.1602426 28508060 PMC5425238

[JCS264887C24] Rhee, H.-W., Zou, P., Udeshi, N. D., Martell, J. D., Mootha, V. K., Carr, S. A. and Ting, A. Y. (2013). Proteomic mapping of mitochondria in living cells via spatially restricted enzymatic tagging. *Science* 339, 1328-1331. 10.1126/science.1230593 23371551 PMC3916822

[JCS264887C25] Richens, J. H., Dmitrieva, M., Zenner, H. L., Muschalik, N., Butler, R., Glashauser, J., Camelo, C., Luschnig, S., Munro, S., Rittscher, J. et al. (2025). MSP-tracker: a versatile vesicle tracking software tool used to reveal the spatial control of polarized secretion in Drosophila epithelial cells. *PLoS Biol.* 23, e3003099. 10.1371/journal.pbio.3003099 40208901 PMC12021295

[JCS264887C26] Roux, K. J., Kim, D. I., Raida, M. and Burke, B. (2012). A promiscuous biotin ligase fusion protein identifies proximal and interacting proteins in mammalian cells. *J. Cell Biol.* 196, 801-810. 10.1083/jcb.201112098 22412018 PMC3308701

[JCS264887C27] Satoh, A. K., O'Tousa, J. E., Ozaki, K. and Ready, D. F. (2005). Rab11 mediates post-Golgi trafficking of rhodopsin to the photosensitive apical membrane of Drosophila photoreceptors. *Development* 132, 1487-1497. 10.1242/dev.01704 15728675

[JCS264887C28] Schlichting, K., Wilsch-Bräuninger, M., Demontis, F. and Dahmann, C. (2006). Cadherin Cad99C is required for normal microvilli morphology in Drosophila follicle cells. *J. Cell Sci.* 119, 1184-1195. 10.1242/jcs.02831 16507588

[JCS264887C29] Stevens, L. M., Zhang, Y., Volnov, Y., Chen, G. and Stein, D. S. (2019). Isolation of secreted proteins from Drosophila ovaries and embryos through in vivo BirA-mediated biotinylation. *PLoS ONE* 14, e0219878. 10.1371/journal.pone.0219878 31658274 PMC6816556

[JCS264887C30] Tay, N. E. S., Ryu, K. A., Weber, J. L., Olow, A. K., Cabanero, D. C., Reichman, D. R., Oslund, R. C., Fadeyi, O. O. and Rovis, T. (2023). Targeted activation in localized protein environments via deep red photoredox catalysis. *Nat. Chem.* 15, 101-109. 10.1038/s41557-022-01057-1 36216892 PMC9840673

[JCS264887C31] Töpfer, U., Guerra Santillán, K. Y., Fischer-Friedrich, E. and Dahmann, C. (2022). Distinct contributions of ECM proteins to basement membrane mechanical properties in Drosophila. *Development* 149. dev200456. 10.1242/dev.200456 35575071

[JCS264887C32] Wang, S., Tan, K. L., Agosto, M. A., Xiong, B., Yamamoto, S., Sandoval, H., Jaiswal, M., Bayat, V., Zhang, K., Charng, W.-L. et al. (2014). The retromer complex is required for Rhodopsin recycling and its loss leads to photoreceptor degeneration. *PLoS Biol.* 12, e1001847. 10.1371/journal.pbio.1001847 24781186 PMC4004542

[JCS264887C33] Westberg, M., Bregnhøj, M., Etzerodt, M. and Ogilby, P. R. (2017). No photon wasted: an efficient and selective singlet Oxygen photosensitizing protein. *J. Phys. Chem. B* 121, 9366-9371. 10.1021/acs.jpcb.7b07831 28892628

[JCS264887C34] Wiater, I., Born, J. G. P. and Louw, R. (2000). Products, rates, and mechanism of the gas-phase condensation of phenoxy radicals between 500-840 K. *Eur. J. Org. Chem.* 2000, 921-928. 10.1002/(SICI)1099-0690(200003)2000:6<921::AID-EJOC921>3.0.CO;2-P

[JCS264887C35] Wu, X., Tanwar, P. S. and Raftery, L. A. (2008). Drosophila follicle cells: morphogenesis in an eggshell. *Sem. Cell Dev. Biol.* 19, 271-282. 10.1016/j.semcdb.2008.01.004 PMC2430523 18304845

[JCS264887C36] Xiong, Z., Lo, H. P., McMahon, K.-A., Martel, N., Jones, A., Hill, M. M., Parton, R. G. and Hall, T. E. (2021). In vivo proteomic mapping through gfp-directed proximity-dependent biotin labelling in zebrafish. *eLife* 10, e64631. 10.7554/eLife.64631 33591275 PMC7906605

[JCS264887C37] Yang, Z., Zhang, Y., Fang, Y., Zhang, Y., Du, J., Shen, X., Zhang, K., Zou, P. and Chen, Z. (2025). Spatial barcoding reveals reaction radii and contact-dependent mechanism of proximity labeling. *Nat. Chem. Biol.*, 1-10. 10.1038/s41589-025-02086-w 41444830

