# Supplementary material for: Radical diffusion, not lifetime, determines the range of peroxidase-based proximity labelling
Source: J Cell Sci. 2026 Jun 15;139(11):jcs264887. doi: 10.1242/jcs.264887 (PMC13327535; doi:10.1242/jcs.264887)
Supplement: Supplementary information [file joces-139-264887-s1.pdf]

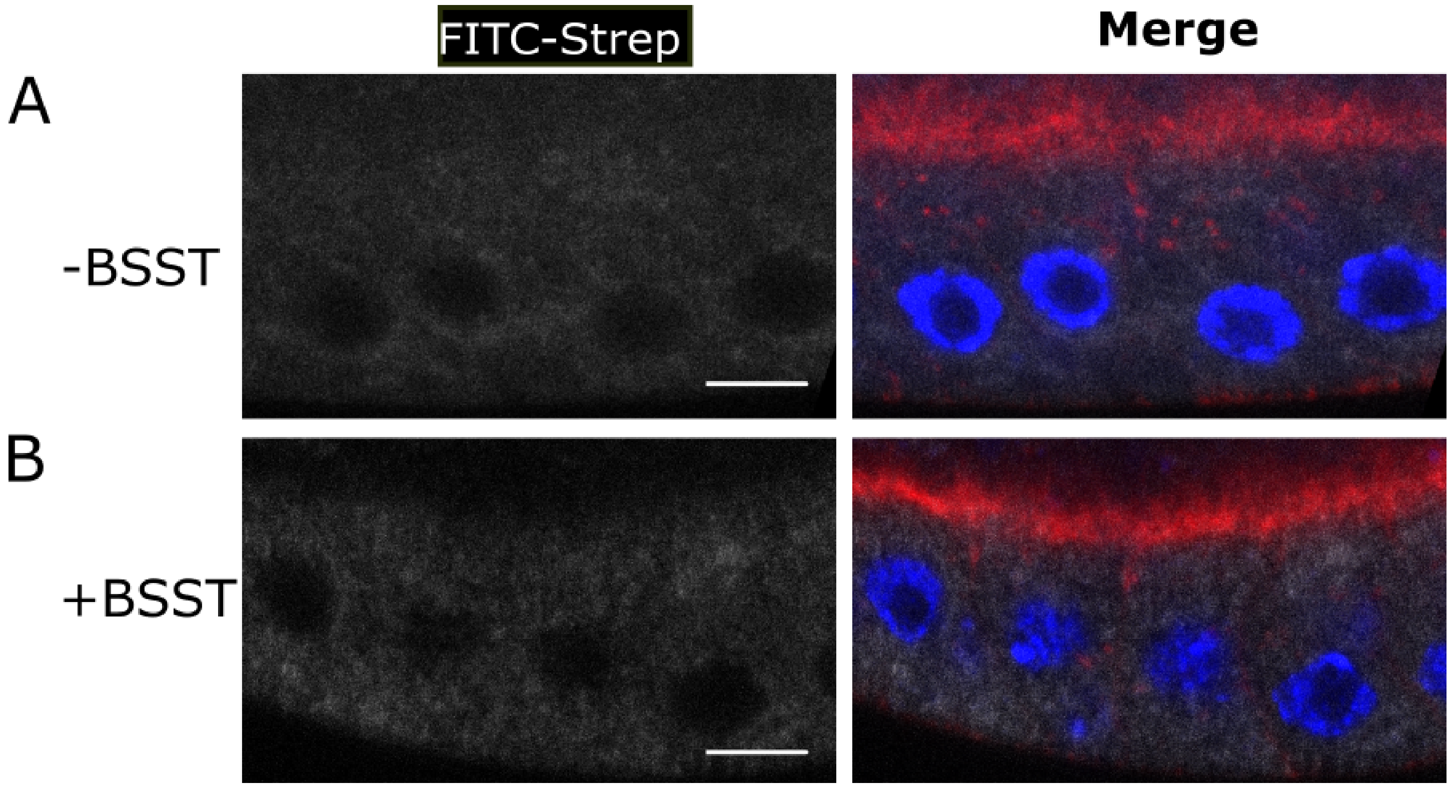

Fig. S1. Biotin-label is necessary but not sufficient for HRP-mediated proximity labelling in follicle cells.

(A) Negative control lacking biotin-SS-tyramide (−BSST) exhibits negligible FITC-Strep signal, confirming that nonspecific streptavidin binding is minimal, and the FITC-Strep signal observed during the labelling reactions is specific. (B) Control ovaries incubated with BSST in the absence of any HRP-tagged cargo (+BSST) show no detectable basal FITC-streptavidin signal, indicating that BSST alone does not induce biotinylation of ECM. This demonstrates that the basal labelling observed under experimental conditions is not due to local radical generation by the ECM but arises from the diffusion of phenoxy radicals produced from HRP-tagged baits. DAPI (blue) stains the nuclei and Phalloidin (red) stains F-actin. N = 3 flies or 6 ovaries stained per condition. The scale bar is 10 µm.

Table S1. Fly lines used in this work

Number	Line	Location	Reference
1	UAST-SBP-APEX2-Cad99c	Attp2	Used in this study
2	UAST-SBP-HRP-Cad99c	Attp2	Used in this study
3	UAST-SBP-HRP-Fas3	Attp2	Used in this study
4	UAST-SBP-HRP-Ndg	Attp2	Used in this study
5	Traffic Jam-Gal4/CyO (PGawBNP1624)	2L	gift from S.Goto
6	w; 2xUAST-SA-KDEL/CyO; UAST-SBP-HRP-Cad99c/TM6B		Used in this study
7	w; 2xUAST-SA-KDEL/CyO; UAST-SBP-HRP-Fas3/TM6B		Used in this study
8	2xUAST-Streptavidin-KDEL/CyO	attP40, attP51C1	gift from S.Luschnig

Journal of Cell Science • Supplementary information

Table S2. Primer Details

Number	Name	Sequence (5'-3')
1	Cad99cSP-EcoRI-SP.F	AGAGAACTCTGAATAGGGAATTGGGATGGCCGCTAGGAACTCAC
2	Cad99c-APEX2-SBP.R	GGTAAGACTTTCCACCTGCAGGTGGTTCACG
3	Cad99c-SBP-APEX2.F	CCACCTGCAGGTGGAAAGTCTTACCCAACTGTGAGT
4	Cad99c-Cad99c-APEX2.R	AACCGCTGCCCGAGGCATCAGCAAACCCAAG
5	Cad99c-APEX2-Cad99c.F	TTTGCTGATGCCTCGGGCAGCGGTTCG
6	Cad99c-STOP-NotI.R	ATCCTCTAGAGGTACCCTCGAGCCGCCTACAGTTCCGTAGTTGTTTCCACCT
7	Cad99c-HRP-SBP.R	TTGGCTAGCACCCTGCAGGTGGTTCACGTTGACCTTG
8	Cad99c-SBP-HRP.F	CGTGAACCACCTGCAGG TGCTAGCCA ACTTACC
9	Cad99c-Cad99c-HRP.R	GCTGCCCGAGGATCCAGAGTTGGAGTTCACCACCCTACAG
10	Cad99c-HRP-Cad99c.F	CTCCAACTCTGGATCCTCGGGCAGCGGTTCGGGCAAGTCGCA
11	Fas3-SP.F	ATAGGGAATTGGGAATTCGTTAACAATGTCACGGATC
12	FAS3-HRP-SBP.R	TAAGTTGGCTAGCACCCTGCAGGTGGTTCACGTTGAC
13	FAS3-SBP-HRP.F	GAACCCCTGCAGG TGCTAGCCAACTTACCCCTACCTT
14	FAS3-FAS3-HRP.R	GGCACTAGTGGATCCGGATCCAGAGTTGGAGTTCACCA
15	FAS3-HRP-FAS3.F	ACTCTGGATCCGGATCCACTAGTGCCCAGGTGAATGTGGAA
16	Fas3-Fas3-STOP.R	TAGAGGTACCCTCGAGCCGCTCATTTTTTTCCTTCGCCTCCCTTTTGCA
17	Ndg-SP. F	ATAGGGAATTGGGAATTCGTTAACAATGTTGCCCTTC
18	Ndg-NDG-STOP.R	CTAGAGGTACCCTCGAGTTAGTAGCCAGGCGCCAGCA
19	NDG-HRP-SBP.R	GTTGGCTAGCGCCACCTGCAGGTGGTTCACGTTGACC
20	NDG-SBP-HRP.F	CCACCTGCAGGTGGCGCTAGCCAACTTACCCCTACCTT
21	NDG-NDG-HRP.R	CTAGATCTCATATGGGATCCGGATCCAGAGTTGGAGTTCA
22	NDG-HRP-NDG.F	GGATCCGGATCCCATATGAGATCTAGGCCTACTAGTCAG

Journal of Cell Science • Supplementary information
