# Supplementary material for: Radical diffusion, not lifetime, determines the range of peroxidase-based proximity labelling
Source: J Cell Sci. 2026 Jun 15;139(11):jcs264887. doi: 10.1242/jcs.264887 (PMC13327535; doi:10.1242/jcs.264887)
Supplement: Supplementary file 1 [file joces-139-264887_review_history.pdf]

Journal of
Cell Science

Radical diffusion, not lifetime, determines the range of peroxidase-based proximity labelling

Samarpita Sen and Daniel St Johnston
DOI: 10.1242/jcs.264887

Editor: Richa Rikhy
Review timeline
Original submission: 17 March 2026
Editorial decision: 20 April 2026
First revision received: 7 May 2026
Accepted: 12 May 2026

Original submission

First decision letter

MS ID#: jcs.264887

MS TITLE: The density of local reactive sites, not radical lifetime, limits the range of peroxidase-based proximity labelling

AUTHORS: Daniel St Johnston; Samarpita Sen

ARTICLE TYPE: Tools and Resources

Dear Dr St Johnston,

We have now reached a decision on the above manuscript.

As you will see, the reviewers gave favourable reports but raised some critical points that will require amendments to your manuscript. I hope that you will be able to carry these out because I would like to be able to accept your paper after the comments are addressed.

Reviewer 1

Advance summary and potential significance to field

This work demonstrates that peroxidase-based proximity labelling range is constrained by local reactive site density, not radical lifetime. Radicals diffuse over several micrometres in epithelia, generating artefactual basal signals. The basement membrane serves as a radical sink. Adjusting substrate concentration or reaction time restores spatial fidelity. These findings challenge the ~20 nm labelling paradigm and offer crucial guidance for robust spatial proteomics in structurally complex tissues.

Comments for the author

Major comments [Please request additional experiments only if they are essential for supporting the conclusions; authors should be encouraged to highlight any claims that are preliminary or speculative, or to discuss any pitfalls or alternative interpretations in a 'Limitations' section]

© 2026. Published by The Company of Biologists under the terms of the Creative Commons Attribution License (https://creativecommons.org/licenses/by/4.0/).

None.

Minor comments

General comments
This manuscript identifies a fundamental constraint in peroxidase-based proximity labeling (PL): the spatial range of labeling is governed by the density of local reactive sites rather than the intrinsic lifetime of phenoxy radicals. The authors demonstrate that under standard conditions, radicals diffuse over several micrometers in Drosophila follicular epithelium, producing artifactual basal labeling even when baits are apical or lateral. They attribute this to the basement membrane acting as a high-capacity sink for radicals, and show that adjusting substrate concentration or reaction time restores spatial fidelity. These findings challenge the widely accepted ~20 nm labeling radius and have direct implications for the design and interpretation of PL experiments in structured tissues.

The data are robust, the controls are appropriate, and the conclusions are supported by multiple lines of evidence. Some clarification of terminology, quantification, and methodological details would enhance precision and reproducibility.

Specific comments
1. The title could be more concise and direct. Consider: Radical Diffusion, Not Lifetime, Determines Peroxidase-Based Proximity Labeling Range.
2. Figure 3 shows clear spatial mismatch between HRP-tagged bait and biotinylation signal. However, the intensity scales are not provided. Adding quantitative intensity profiles across the apical-basal axis would support the claim of long-range diffusion more objectively.
3. The conclusion that the basement membrane acts as a radical sink is central. Yet, the current data do not exclude contributions from other extracellular factors. A brief discussion of whether other ECM components or secreted proteins could play a similar role would clarify the specificity of the proposed mechanism.
4. In Figure 4, the shift from basal to apical/lateral labeling upon dilution of BSST or shortening reaction time is convincing but lacks statistical assessment. Including error bars or significance indicators would strengthen the conclusion that these changes restore specificity.
5. The Methods describe optimisation of BSST concentration but do not detail the criteria used to define "optimal." Specifying whether this was based on spatial recovery, signal-to-noise ratio, or reproducibility would aid reproducibility.
6. The model in Figure 6A illustrates radical diffusion and ECM interaction but does not define the basis for estimating diffusion distances (e.g., Fick's law, empirical measurements). A brief note on the calculation or supporting literature would improve mechanistic clarity.
7. The Discussion appropriately highlights the need for titration of radical production. Providing a concise set of practical guidelines (e.g., recommended BSST concentration ranges, reaction time limits) would translate the findings into actionable advice for users of PL technologies.

Reviewer 2

Advance summary and potential significance to field

This research paper investigates technical limitations and improvements for peroxidase-based proximity labelling, a method used to map the interactome of specific proteins. While the technique has been reported to label only proteins within a 20 nm labelling radius, the authors discovered that biotin-phenoxy radicals can actually diffuse over several microns in Drosophila epithelial tissues. This wide diffusion causes a misleading basal biotinylation signal because the basement membrane acts as a high-capacity sink for reactive radicals. The study proposes that this spatial inaccuracy is caused by an excess of radicals relative to available local targets, rather than the radical's natural lifespan. To solve this, the researchers successfully restored spatially faithful labelling by reducing the concentration of biotin-phenol or shortening the reaction times. Ultimately, the findings highlight that achieving accurate results requires careful optimization of reagent levels or incubation times.

© 2026. Published by The Company of Biologists under the terms of the Creative Commons Attribution License (https://creativecommons.org/licenses/by/4.0/).

The study also introduces as an improvement to the peroxidase-based proximity ligation methodology the use of Biotin-SS-Tyramide (BSST) instead of Biotin-Phenol (BP) to reduce background from endogenously biotinylated proteins.

Overall, this study is important and of interest to an audience investigating protein-protein interactions as it points to potential artifacts and the need to titrate carefully the amount and duration of biotin labeling in proximity ligation experiments. However, while the main message of the paper is clear, some of the assays used are not state-of-the-art and could have easily been complemented by more sophisticated methods. In particular, the use of silver-stained protein gels instead of mass spectrometry data to demonstrate the differences between BSST and BP labeling and the results of HRP-C99C versus HRP-Ndg proximity labeling is insufficient. My impression was that the authors wanted to publish this study as an independent paper in addition to a second, main paper describing the molecular composition of the apical, lateral and basal proteome. Thus, in my opinion the study as is would deserve being published but is not of sufficient general interest to be published in JCS. This would be different if the mass spec data would be added together with a discussion of the differences in the apical, lateral and basal proteomes.

Comments for the author

Major comments [Please request additional experiments only if they are essential for supporting the conclusions; authors should be encouraged to highlight any claims that are preliminary or speculative, or to discuss any pitfalls or alternative interpretations in a 'Limitations' section]
* Figure 2C and Fig. 6B, C: The analysis of biotinylated proteins from proximity ligation experiments is routinely done by mass spectrometry to identify the labeled proteins. I find this mandatory if the authors want this study to be published in JCS.
* Discussion, page 18, last paragraph: Here, alternative approaches for proximity labeling are discussed, including TurboID. What is missing in my opinion here is a statement regarding the different mechanism of HRP-mediated labeling compared to biotin labeling mediated by BirA-related biotin ligases, which does not rely on diffusion of phenoxy radicals.

Minor comments

First revision

<u>Author response to reviewers' comments</u>

General comments

This manuscript identifies a fundamental constraint in peroxidase-based proximity labeling (PL): the spatial range of labeling is governed by the density of local reactive sites rather than the intrinsic lifetime of phenoxy radicals. The authors demonstrate that under standard conditions, radicals diffuse over several micrometers in Drosophila follicular epithelium, producing artifactual basal labeling even when baits are apical or lateral. They attribute this to the basement membrane acting as a high-capacity sink for radicals, and show that adjusting substrate concentration or reaction time restores spatial fidelity. These findings challenge the widely accepted ~20 nm labeling radius and have direct implications for the design and interpretation of PL experiments in structured tissues.

The data are robust, the controls are appropriate, and the conclusions are supported by multiple lines of evidence. Some clarification of terminology, quantification, and methodological details would enhance precision and reproducibility.

Specific comments
1. The title could be more concise and direct. Consider: Radical Diffusion, Not Lifetime, Determines Peroxidase-Based Proximity Labeling Range

© 2026. Published by The Company of Biologists under the terms of the Creative Commons Attribution License (https://creativecommons.org/licenses/by/4.0/).

We have changed the title as requested.

2. Figure 3 shows clear spatial mismatch between HRP-tagged bait and biotinylation signal. However, the intensity scales are not provided. Adding quantitative intensity profiles across the apical-basal axis would support the claim of long-range diffusion more objectively.

We thank the reviewer for this helpful suggestion. To quantitatively assess the spatial relationship between the HRP-tagged cargo and the resulting biotinylation signal through FITC-Strep labelling, we have now included intensity line profiles across the apical-basal axis of follicle cells (new Fig. 3D). These analyses reveal a clear spatial offset between the HRP signal and the biotinylation signal for apical (HRP-Cad99c) and lateral (HRP-Fas3) cargoes, with the peak biotinylation intensity occurring at the basal surface for both. In contrast, the basal bait (HRP-Ndg) shows strong spatial overlap between the two signals. We further quantified this displacement by measuring the distance between the peak intensities of the HRP and biotinylation signals, confirming a several-micron separation between HRP-C99c/HRP-Fas3 and FITC-Strep peaks, consistent with long-range diffusion. These are now included in the Results section. All intensity measurements were performed on raw images without rescaling.

3. The conclusion that the basement membrane acts as a radical sink is central. Yet, the current data do not exclude contributions from other extracellular factors. A brief discussion of whether other ECM components or secreted proteins could play a similar role would clarify the specificity of the proposed mechanism.

While our data support the idea that the basement membrane as the major sink for diffusing phenoxy radicals, we cannot exclude the possibility that other extracellular proteins also contribute to radical capture. In principle, any protein enriched in electron-rich amino acids could serve as a potential target for phenoxy radicals and this might include secreted proteins in the extracellular space. However, our egg chambers are cultured ex vivo in Schneider's medium supplemented with Insulin. Any secreted proteins in the fly haemolymph will be diluted by the medium during culture. The only protein in the medium is Insulin, which also contains peptides derived from yeast (Yeastolate), but neither of these is likely to act as a basal sink, since they are not confined basally and can diffuse laterally. Our observations therefore suggest that the basement membrane is likely to be the dominant sink. In support of this view, enzymatic disruption of the basement membrane by collagenase treatment leads to a marked reduction in the basal signal and reveals the underlying apical labelling, consistent with loss of a high-capacity sink. Together, these observations suggest that while radical scavenging is not unique to the basement membrane, its high protein density and biochemical composition make it a particularly effective sink for diffusing phenoxy radicals in the follicular epithelium.

4. In Figure 4, the shift from basal to apical/lateral labeling upon dilution of BSST or shortening reaction time is convincing but lacks statistical assessment. Including error bars or significance indicators would strengthen the conclusion that these changes restore specificity.

We have now quantified the shift from basal to apical or lateral labelling by measuring the apical-to-basal and lateral-to-basal fluorescence intensity ratio of the FITC-Streptavidin signal as the BSST concentration or reaction time is changed. This analysis demonstrates that the strong basal enrichment observed under standard conditions is significantly reduced upon BSST dilution or shorter reaction times. The quantification with error bars and statistical analysis is now included in Fig 4.

5. The Methods describe optimisation of BSST concentration but do not detail the criteria used to define "optimal." Specifying whether this was based on spatial recovery, signal-to-noise ratio, or reproducibility would aid reproducibility.

We have now clarified the criteria used to define optimal labelling conditions in the Methods section. Briefly, optimal BSST concentrations were selected based on the restoration of spatial specificity, quantified as the ratio of signal in the expected membrane domain relative to the basal domain, together with sufficient signal intensity and reproducibility across samples.

© 2026. Published by The Company of Biologists under the terms of the Creative Commons Attribution License (https://creativecommons.org/licenses/by/4.0/).

6. The model in Figure 6A illustrates radical diffusion and ECM interaction but does not define the basis for estimating diffusion distances (e.g., Fick's law, empirical measurements). A brief note on the calculation or supporting literature would improve mechanistic clarity.

We thank the reviewer for this suggestion. We have now clarified the basis for estimating diffusion distances in the Discussion. Specifically, we explicitly relate our estimates to classical diffusion theory ($\langle x^2 \rangle = 6Dt$), using literature values for diffusion coefficients and radical lifetimes. This addition provides a mechanistic framework that supports the micron-scale diffusion inferred from our experimental observations. We have added the following text to the discussion:

"The root mean square displacement of a diffusing molecule is given by $\langle x^2 \rangle = 2Dt$ (in one dimension) or 6Dt (in three dimensions), where D is the diffusion coefficient and t is the lifetime of the species. Using the estimated diffusion coefficient for biotin-phenol radicals of 200μm s^{-1} (Yang et al. 2025) and lifetimes in the low millisecond range, this yields diffusion distances in the range of 0.6-1.0μm, which is much larger than the 20-300 nm reported by Rhee et al. (2013) and Oakley et al. (2022), but significantly smaller than the distance from the apical membrane to the basal membrane of the follicle cells, which is 5-8μm. This suggests that the half-life of phenoxy radicals depends on their probability of reacting with an electron-rich target, and once the local targets are saturated, they can continue diffusing for up to 50 msecs until they react."

7. The Discussion appropriately highlights the need for titration of radical production. Providing a concise set of practical guidelines (e.g., recommended BSST concentration ranges, reaction time limits) would translate the findings into actionable advice for users of PL technologies.

We have now added a concise set of practical guidelines in the Discussion outlining recommended ranges for BSST concentration, reaction times, and validation strategies. These guidelines are based on our qualitative and quantitative analyses and are intended to help users optimize proximity labelling conditions in their specific contexts.

" Based on these findings, we propose several practical guidelines for peroxidase-based proximity labelling in epithelial tissues. First, biotin-phenol or biotin-SS-tyramide concentrations should be empirically titrated, as standard concentrations (e.g., ~500 μM) can lead to extensive diffusion and loss of spatial specificity. In our system, concentrations in a much lower range (10-40 μM) were sufficient to restore domain-restricted labelling while maintaining detectable signal. Second, reducing the duration of the labelling reaction can similarly improve spatial resolution; shortening reaction times (e.g., from 1 min to 15 s) significantly limited diffusion. Third, optimisation should be guided by image-based validation, ensuring that the biotinylation signal matches the known localization of the bait protein. Finally, tissues with abundant extracellular matrices or high densities of reactive residues may require more stringent control of radical production, as these environments can act as high-capacity sinks for diffusing radicals. Together, these considerations provide a practical framework for optimizing proximity labelling conditions to achieve spatially faithful proteomic mapping."

Reviewer 2: SUMMARY OF THE ADVANCE MADE IN THIS PAPER AND ITS POTENTIAL SIGNIFICANCE TO THE FIELD

This research paper investigates technical limitations and improvements for peroxidase-based proximity labelling, a method used to map the interactome of specific proteins. While the technique has been reported to label only proteins within a 20 nm labelling radius, the authors discovered that biotin-phenoxy radicals can actually diffuse over several microns in Drosophila epithelial tissues. This wide diffusion causes a misleading basal biotinylation signal because the basement membrane acts as a high-capacity sink for reactive radicals. The study proposes that this spatial inaccuracy is caused by an excess of radicals relative to available local targets, rather than the radical's natural lifespan. To solve this, the researchers successfully restored spatially faithful labelling by reducing the concentration of biotin-phenol or shortening the reaction times. Ultimately, the findings highlight that achieving accurate results requires careful optimization of reagent levels or incubation times.

© 2026. Published by The Company of Biologists under the terms of the Creative Commons Attribution License (https://creativecommons.org/licenses/by/4.0/).

The study also introduces as an improvement to the peroxidase-based proximity ligation methodology the use of Biotin-SS-Tyramide (BSST) instead of Biotin-Phenol (BP) to reduce background from endogenously biotinylated proteins.

Overall, this study is important and of interest to an audience investigating protein-protein interactions as it points to potential artifacts and the need to titrate carefully the amount and duration of biotin labeling in proximity ligation experiments. However, while the main message of the paper is clear, some of the assays used are not state-of-the-art and could have easily been complemented by more sophisticated methods. In particular, the use of silver-stained protein gels instead of mass spectrometry data to demonstrate the differences between BSST and BP labeling and the results of HRP-C99C versus HRP-Ndg proximity labeling is insufficient. My impression was that the authors wanted to publish this study as an independent paper in addition to a second, main paper describing the molecular composition of the apical, lateral and basal proteome. Thus, in my opinion the study as is would deserve being published but is not of sufficient general interest to be published in JCS. This would be different if the mass spec data would be added together with a discussion of the differences in the apical, lateral and basal proteomes.

SUGGESTIONS TO AUTHORS

Major comments [Please request additional experiments only if they are essential for supporting the conclusions; authors should be encouraged to highlight any claims that are preliminary or speculative, or to discuss any pitfalls or alternative interpretations in a 'Limitations' section]

* Figure 2C and Fig. 6B, C: The analysis of biotinylated proteins from proximity ligation experiments is routinely done by mass spectrometry to identify the labeled proteins. I find this mandatory if the authors want this study to be published in JCS.

We agree that mass spectrometric analysis is the best approach for identifying biotinylated proteins in proximity labelling experiments, but that was not the goal of our study. Instead, we set out to optimise conditions to ensure that labelling is proximal to the peroxidase tagged bait protein. This requires measuring the location, number and yield of biotinylated proteins, but their identities are not important. Silver staining of SDS PAGE gels is a more efficient and much cheaper way of analysing the number and yield of biotinylated proteins this for several reasons. 1) Silver staining should reveal all the labelled proteins, whereas mass-spectroscopy only identifies some of the proteins, depending on whether they are solubilized, efficiently digested by Trypsin, are not too glycosylated and contain peptides that are detectable on the mass-spectrometer. It is particularly challenging to identify membrane proteins by mass-spectroscopy, because they are often heavily glycosylated and need to be extracted from the plasma membrane with detergents, which must then be removed before digesting them with Trypsin and loading them on the mass -spec, and these additional steps often lead to low yields or insolubility. 2) Normal label-free mass-spectroscopy is at best semi-quantitative, whereas silver staining gives a better idea of the relative abundance of the biotinylated proteins. 3) One of the two silver-stained gels that we show in the manuscript (Fig 2C) shows the background of labelled proteins in control samples. Since we were not interested in the identities of these proteins, but in the relative number of background bands when labelling with Biotin-Phenol or BSST, it did not seem worth the extra time and expense of performing mass-spectroscopy.

* Discussion, page 18, last paragraph: Here, alternative approaches for proximity labeling are discussed, including TurboID. What is missing in my opinion here is a statement regarding the different mechanism of HRP-mediated labeling compared to biotin labeling mediated by BirA-related biotin ligases, which does not rely on diffusion of phenoxy radicals.

We thank the reviewer for this helpful suggestion. We have now clarified the mechanistic differences between peroxidase-based proximity labelling and biotin ligase-based approaches such as TurboID in the Discussion. In particular, we highlight that HRP/APEX2 methods rely on diffusible radical intermediates, whereas ligase-based methods may involve direct transfer of activated biotin onto proteins in their immediate vicinity and may therefore be less subject to diffusion-driven artefacts.

© 2026. Published by The Company of Biologists under the terms of the Creative Commons Attribution License (https://creativecommons.org/licenses/by/4.0/).

" Peroxidase-based methods such as HRP and APEX2 generate diffusible phenoxy radicals, which can travel considerable distances before reacting with target proteins. As a result, the effective labelling radius is strongly influenced by radical lifetime, local target density, and diffusion constraints, as demonstrated in this study. It has recently been proposed that the biotin-AMP intermediate produced by proximity labelling strategies based on engineered biotin ligases, such as BioID and TurboID remains associated with the biotin ligase for up to 10 seconds and is directly transferred to nearby lysine residues, without the involvement of a freely diffusible radical species (Yang et al, 2025). Consequently, ligase-based approaches may be more spatially constrained and less susceptible to diffusion-driven artefacts, although they are limited by slower kinetics and a requirement for sustained protein proximity."

Second decision letter

MS ID#: jcs.264887R1

MS Title: Radical diffusion, not lifetime, determines the range of Peroxidase-based proximity labelling

Authors: Daniel St Johnston; Samarpita Sen

Article Type: Tools and Resources

Dear Dr St Johnston,

I am happy to tell you that your manuscript has been accepted for publication in Journal of Cell Science, pending standard publication integrity checks.

© 2026. Published by The Company of Biologists under the terms of the Creative Commons Attribution License (https://creativecommons.org/licenses/by/4.0/).
